# Considerations of Antibody Geometric Constraints on NK Cell Antibody Dependent Cellular Cytotoxicity

**DOI:** 10.3389/fimmu.2020.01635

**Published:** 2020-07-30

**Authors:** Charles D. Murin

**Affiliations:** Department of Integrative Structural and Computational Biology, Scripps Research, La Jolla, CA, United States

**Keywords:** antibody, ADCC, NK cell, structural biology, antibody therapeutics, immune synapse, antibody effector functions, immune signaling

## Abstract

It has been well-established that antibody isotype, glycosylation, and epitope all play roles in the process of antibody dependent cellular cytotoxicity (ADCC). For natural killer (NK) cells, these phenotypes are linked to cellular activation through interaction with the IgG receptor FcγRIIIa, a single pass transmembrane receptor that participates in cytoplasmic signaling complexes. Therefore, it has been hypothesized that there may be underlying spatial and geometric principles that guide proper assembly of an activation complex within the NK cell immune synapse. Further, synergy of antibody phenotypic properties as well as allosteric changes upon antigen binding may also play an as-of-yet unknown role in ADCC. Understanding these facets, however, remains hampered by difficulties associated with studying immune synapse dynamics using classical approaches. In this review, I will discuss relevant NK cell biology related to ADCC, including the structural biology of Fc gamma receptors, and how the dynamics of the NK cell immune synapse are being studied using innovative microscopy techniques. I will provide examples from the literature demonstrating the effects of spatial and geometric constraints on the T cell receptor complex and how this relates to intracellular signaling and the molecular nature of lymphocyte activation complexes, including those of NK cells. Finally, I will examine how the integration of high-throughput and “omics” technologies will influence basic NK cell biology research moving forward. Overall, the goal of this review is to lay a basis for understanding the development of drugs and therapeutic antibodies aimed at augmenting appropriate NK cell ADCC activity in patients being treated for a wide range of illnesses.

## Introduction

Antibodies have a bifunctional role within the immune system. This role is physically built into their structure through two parts: the fragment antigen binding (Fab), for recognizing antigen, and the fragment crystallizable (Fc), for recruiting effector immune cells. The process by which antibody-coated cells direct effector cells to attack and kill an opsonized target is known as antibody dependent cellular cytotoxicity (ADCC). This is accomplished through ligation with Fc gamma receptors (FcγRs), which forms a conduit of communication between the target cell (TC) and immune effector cell (1). The FcγRs are an assortment of transmembrane receptors expressed to varying levels on primarily innate, but also some adaptive, immune cells (2). The ability of antibodies to recruit ADCC is a highly desirable trait for therapeutic and vaccine development, and NK cells are of central focus due to their proclivity for ADCC and as a front-line defense immune cell (3–6). While our understanding of antigen-antibody recognition and Fc-FcγR interaction are each quite extensive in isolation, there is still a gap in knowledge about how these two important aspects of antibodies interplay, especially *in vivo*. Combined with frequent incongruency between available *in vitro* and *in vivo* data regarding antibody effector function as well as the generally complicated nature of the human immune system, we are left with a looming question: what makes an effective antibody for recruiting NK cell ADCC?

Answering the question above requires a much better understanding of the underlying molecular basis of antibody and cellular effector functions. A good place to start is at the point of initial contact between an NK cell and TC, known as the immune synapse (IS). This is the point where activating receptors on the NK cell surface bind to the Fc domain of antigen-engaged antibodies and initialize a cascade of events that lead to NK cell activation and ultimately target-cell death. Extensive studies of the T cell receptor have provided valuable insight into the organization of the T cell IS (7–10), but much less is known about the NK cell immune synapse (NKIS).

Antibodies are necessary for clustering activating receptors in the early stages of ADCC. Structural biology has been instrumental in providing a much more detailed view of this initial interaction of antibody and antigen, especially in the context of viral antigens from HIV, influenza and ebolavirus. Depending on the location of antibody epitopes, the Fc domain of the antibody can differ vastly in how it is presented to a surveying NK cell. Many other variables, including antigen shape, size, and density as well as lipid environment and mobility, can also affect Fc presentation. Further, all these variables can change with antibody isotype, subclass and glycosylation as well as FcγR isotype, cellular subclass, FcγR expression and diversity as well as FcγR glycosylation and alleles (2).

With an increasing number of antibody therapeutics, vaccines and immunotherapies entering the clinical market (11), a greater understanding of NK cell mediated ADCC will guide precision medicine and create more effective drugs. In this review, I will focus on current efforts to understand NK cell ADCC, with a particular focus in the context of virally infected cells. I will explore how advances in microscopy techniques as well as the increasing accessibility of big data technologies such as transcriptomics, proteomics, and metabolomics are challenging our understanding of classical immunology and paving a way to fill the gap between *in vitro* and *in vivo* observations. Such advances will reveal new avenues for vetting therapeutics with the greatest chance of success in patients.

## Receptors and Ligands Involved in ADCC

Humans employ an arsenal of a FcγRs that specifically recognize antibodies via their Fc domains (1, 2, 12). These receptors can be inhibitory or activating for the cells on which they reside, denying or providing the initial spark to perform antibody-based effector functions, respectively. While NK cells almost exclusively utilize a single type of activating FcγR (13, 14), it is important to understand the function of FcγRs more broadly. In this section, I will briefly discuss what is currently known about the receptors and ligands involved in ADCC as well as how their interplay differs among peripheral and tissue resident NK cells.

### The FcγRs and Their Antibody Ligands

Each antibody isotype has its own unique Fc receptor, and these have been studied extensively and reviewed elsewhere (1, 12, 15). The receptors include Fc alpha receptor I (FcαRI or CD89) for immunoglobulin (Ig) A (16–18), Fc epsilon receptor I (FcεRI) for IgE (19–21), FcγR for IgG (1, 12) and Fc mu receptor (FcμR) for IgM (22, 23). There is also mixed evidence of a putative receptor for soluble IgD, named Fc delta receptor (Fc∂R) (24, 25). There are additional Ig receptors that reside on other cell types, including the neonatal Fc receptor (FcRn) with a function in recycling antibodies (26, 27), the mixed Fc alpha/mu receptor (Fcα/μR) with a function in endocytosis of IgA/IgM coated microbes (28) and the polymeric Ig receptor with a function in the endocytosis of polymeric IgA and immune complexes (pIgR) (29, 30). Not all antibodies bind to their cognate receptors with equal affinity (31) and each receptor has a unique control over the immune response.

**Figure 1:**
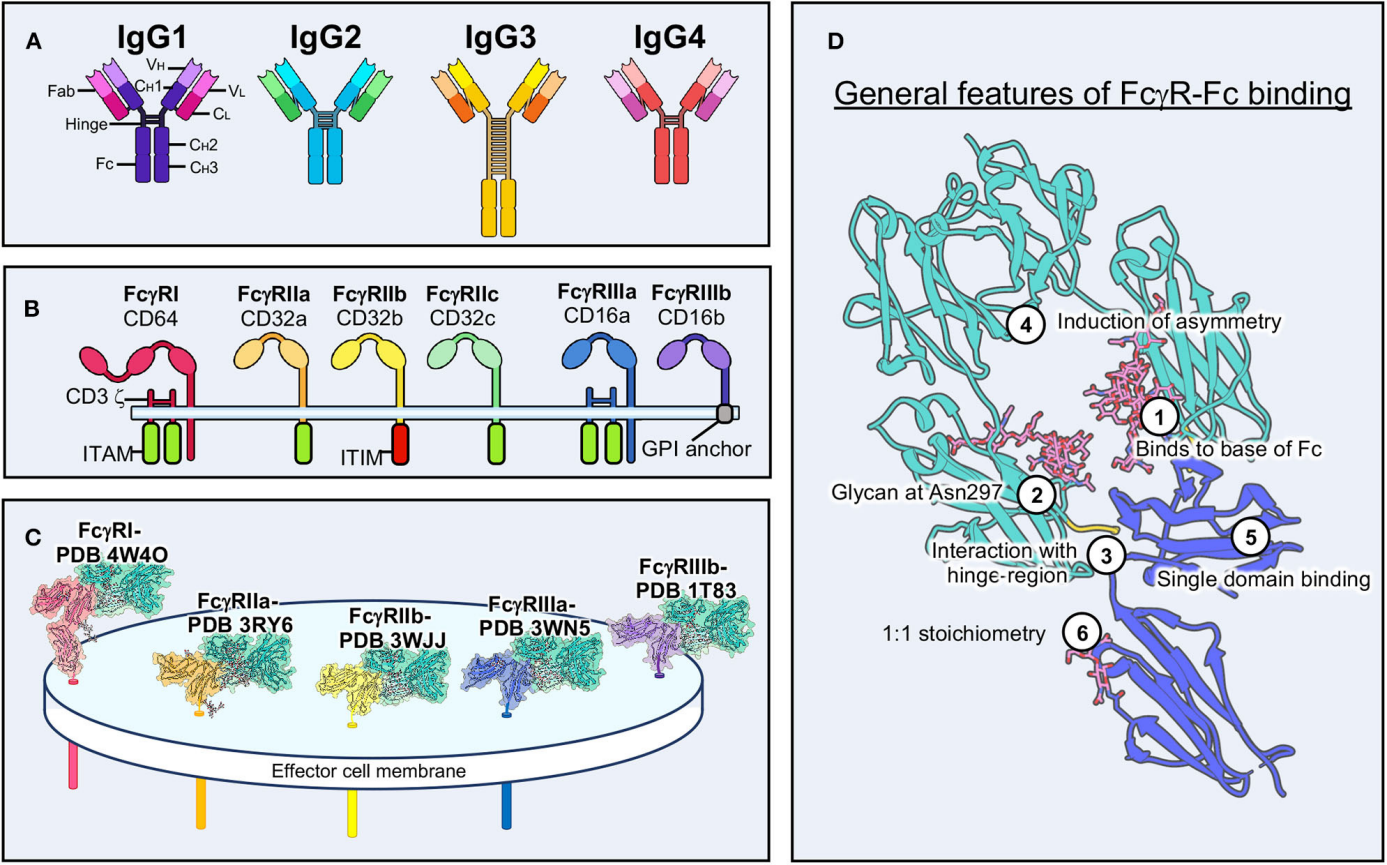
Structural characteristics of antibody-FcγR interactions. **(A)** Schematic of the four IgG antibody isotypes. IgG domains and sub-domains are labeled on the far left. **(B)** Schematic of the six human FcγRs and associated signaling motifs. **(C)** Structural examples of the five main human FcγRs bound to Fc domains with transmembrane domains modeled in the effector cell membrane. **(D)** Conserved features of FcγR-Fc binding. The example shown is of FcγRIIIa (PDB 3WN5). ITAM, immunoreceptor tyrosine-based activation motif; ITIM, immunoreceptor tyrosine-based inhibitory motif; GPI, glycosylphosphatidylinositol.

Most antibody therapeutics are overwhelmingly of the IgG class, which is the primary type of antibody formed in response to vaccines and pathogenic threats (32, 33). IgG also makes up a significant portion of the antibodies in human sera to assist the innate immune response in identifying immediate threats and assisting the adaptive memory response. IgGs exist in four known subclasses in humans, including IgG1, IgG2, IgG3, and IgG4 (Figure 1A). There are six known IgG receptors, including FcγRI (or CD64), FcγRIIa/b/c (or CD32), and FcγRIIIa/b (or CD16) (Figure 1B), and they each display differential binding affinity for these subclasses of IgG (1, 2, 31). Most of FcγRs are activating, signaling through immunoreceptor tyrosine-based activation motifs (ITAMs), with the exception of FcγRIIb, which is an inhibitory receptor and signals through an immunoreceptor tyrosine-based inhibitory motif (ITIM). FcγRIIIa is the most abundant and important receptor on NK cells for inducing ADCC, and is a prototypic cell marker for mature NK cells in the periphery (34–36). While all the IgGs can bind to FcγRIIIa, IgG1 and IgG3 are the most effective at activating NK cells for ADCC (2, 31). FcγRI has the highest affinity for IgG, particularly IgG1 and IgG3, but is not reported to be found on NK cells (1). Interestingly, there are glycan variants of FcγRIIIa that display affinities close to FcγRI, as I will discuss below (37).

Structural biology has been important in elucidating the molecular nature of the FcγR-Fc interaction and examples exist of every human FcγR both liganded to Fc and unliganded (Figure 1C) (38). FcγRs are quite small and are therefore almost exclusively studied by crystallography (12). Small proteins (<100 kDa) are still difficult targets for cryo-electron microscopy (EM) but are becoming increasingly approachable as technology improves (39–41). These receptors adopt an Ig-like fold, similar to antibodies, with two Ig lobes separated by a short elbow (12). Notably, FcγRI has an additional Ig domain, although the function of the third domain is unclear (42).

These structures have shown striking conservation in how IgGs bind to FcγRs (Figure 1D). The majority of molecular interactions occur near the hinge-region of IgG, near the base of the Fc, and are heavily reliant upon a glycan at Asn 297 in the Fc domain (1, 42–44). Binding induces asymmetry within the Fc through interaction with a single domain of FcγRs. Despite Fc domains having two equivalent binding sites for FcγRs, binding to IgGs is monovalent, due to this induced asymmetry. The 1:1 stoichiometry of binding is the same for other FcRs, except for FcαR, which is capable of binding IgA as a dimer (18).

IgG glycosylation can take on many different forms and has major implications for the immune response (45, 46). Afucosylated forms of IgG, for example, are capable of a superior ADCC phenotype and structural evidence indicates that this form of IgG allows for a stronger interaction with FcγRIIIa (47–49). FcγRs are themselves glycosylated to varying degrees (50). Glycosylation is often overlooked in the structural context, due to limitations of crystallography, but has a notable influence on activation and affinity and continues to be explored (37).

### Cellular and Tissue Distribution of FcγRs

**Figure 2:**
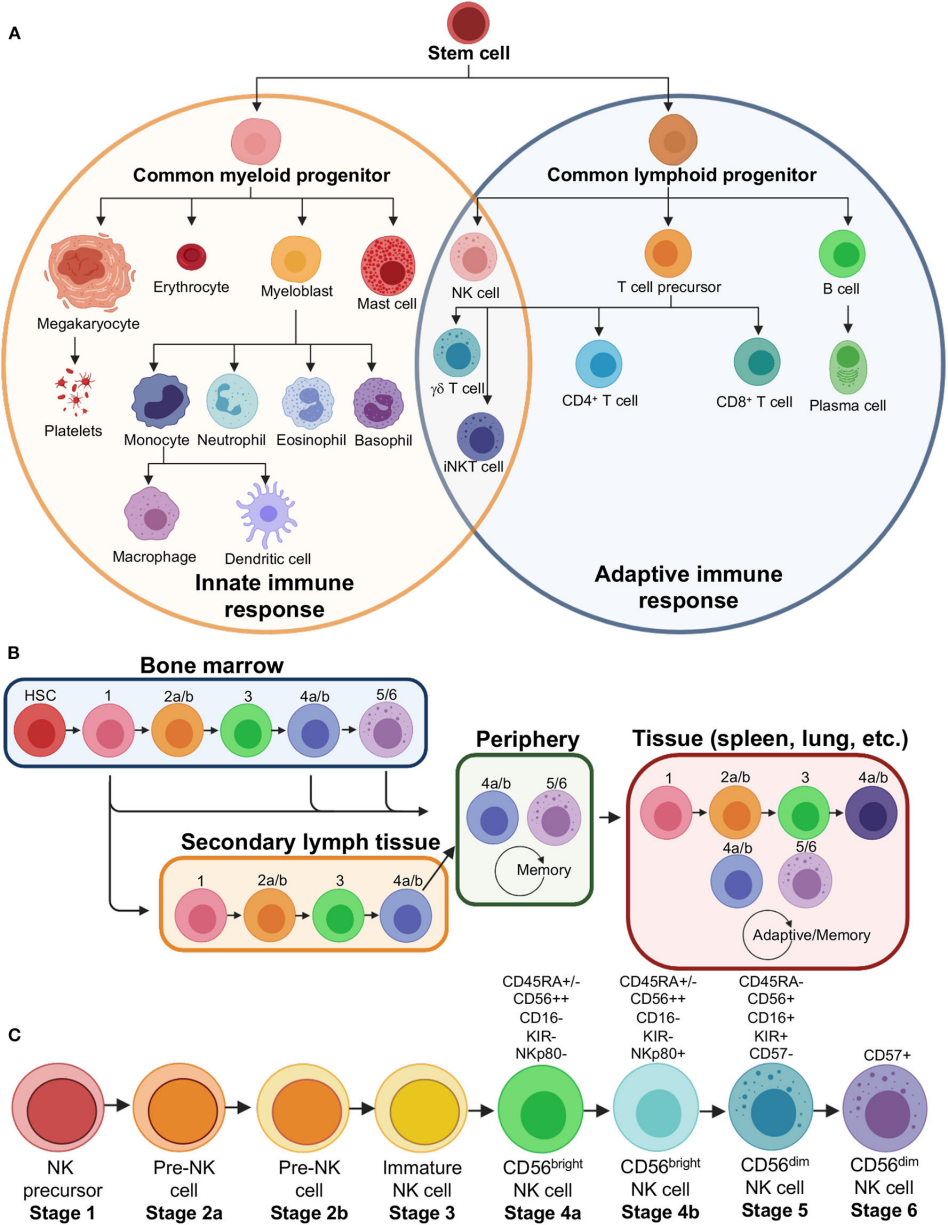
Natural killer cell lineage and development. **(A)** Immune effector cell lineages showing that NK cells derive from a common lymphoid progenitor related to B and T cells, that make up a majority of the adaptive immune response. NK cells, however, share several similarities in function and phenotype to myeloid progenitor cells that make up a majority of the innate immune response. **(B)** Flow chart showing the modern theory of NK cell development, which demonstrates that NK cells may leave bone marrow at various stages and continue development into specialized subsets in the secondary lymph tissue, peripheral blood or become tissue resident NK cells. **(C)** Stages of NK cell development, including distinct sub-stages, with major markers that distinguish mature cell types indicated. HSC, hematopoietic stem cell; iNKT, invariant natural killer T cell.

FcγRs exist mainly on immune cells, but have also been found in some neural cells, liver cells and even as part of viral and bacterial defense mechanisms (51). In terms of immunity, the FcγRs clearly dominate in innate immune cells, likely due to their role in first-line defense and surveillance (Figure 2A). Conversely, there is little evidence for constitutive FcγR expression within adaptive immune cells such as T cells (although some small subset may express FcγRIIIa) and only the presence of the inhibitory receptor FcγRIIb on B cells (1, 51). Nevertheless, FcγR activation by antibodies can recruit the adaptive immune response and other innate cells, and thus ties both arms of the immune system together (Figure 2A). Within the innate cell repertoire, macrophages, monocytes, granulocytes, dendritic cells, and mast cells all express varying combinations of the FcγRs (1, 51). The expression of some receptors can be induced in certain cellular populations, although typically at low levels, or may exist only in a subset of cellular populations. This range of FcγR expression on immune cells is not well-understood but may serve as an advantage to the immune system in being able to quickly respond to a diverse array of insults.

NK cells form a unique cellular subset since they are of the lymphoid lineage, more closely related to B and T cells, but act more like an innate immune cell in their function, are therefore often referred to as innate lymphoid cells or ILCs (Figure 2A) (52–54). NK cells are exceptionally diverse, and I will briefly discuss both their presence in peripheral blood (PB) and tissues (Figure 2B) (55–59). NK cells form a smaller fraction of lymphocytes within the PB but can vary widely from 5 to 20% or even higher depending on the individual. Typically, peripheral NK cells are defined by a lack of CD3 to distinguish them from T cells, a lack of CD19 to distinguish them from B cells, and the presence of CD45 to distinguish them as lymphocytes. Further, NKs are confirmed by the presence of CD56 and CD16 to varying degrees, leading to so called CD56^bright^/CD16^lo/−^ and CD56^dim^/CD16^+^ populations (Figure 2C) (60, 61). CD56^dim^ NK cells are thought to be the cellular population that is best at performing ADCC due to a higher constitutive expression of CD16. This makes CD56^dim^ NK cell lines, such as NK-92 cells, particularly desirable for NK cell engineering and use in *in vitro* ADCC assays (62, 63). CD56^bright^ NK cells can respond rapidly to produce cytokines and chemokines in conjunction with the response of other activated cells, including T cells, dendritic cells and monocytes.

The diversity of NK cells extends to tissue resident NK cells (Figure 2B) (34, 57, 64). CD56^dim^ NK cells, which predominate the ADCC response in PB, are not found ubiquitously in all tissues and may actually be outnumbered by CD56^bright^ cells overall in the human body (57, 64). The population of CD56^dim^ cells capable of ADCC largely exist in the bone marrow as well as lung, spleen, breast and subcutaneous adipose tissue (57). NK cell diversity is extended by varying degrees of chemokine receptors as well as a huge variety of killer immunoglobulin-like receptors (KIRs) (65). This plasticity specializes NK cells to their environment and makes them functionally distinct (Figure 2B). Further, certain NK cells may even develop memory, similar to adaptive immune cells (66, 67).

### Limits to Our Current Understanding of Antibody-FcγR Interactions

There are many gaps that prevent a full understanding of antibody-FcγR interactions. First, all of our molecular observations of antibody-FcγR interactions come from fragments. For example, in every structure of FcγRs, the IgG is severed from the antigen recognition domains (Figure 1C). Conversely, in every structure of antibody bound to antigen, the Fc fragment is missing. While there are a few structures of IgGs alone, as well as biophysical characterization that demonstrate their range of flexibility and overall architecture, this flexibility has largely restricted their study in complexes due to historical limitations in structural biology (68).

Next, FcγRs are after all membrane glycoproteins, but there are no structures of the full-length receptors. Additionally, many activating receptors use transmembrane adaptors that are necessary for cell surface expression and signaling, proteins that have only limited structural observation in isolation (14, 69, 70). While FcγRs are mostly single pass transmembrane proteins (with the exception of FcγRIIIb, which is a GPI-anchored protein and whose signaling is not well-understood), there are almost certainly higher order assemblies that must form in order for signaling to proceed.

Finally, known molecular observations have not yet been reconciled with the crowded but organized environment of the IS. Most of our understanding of the NKIS has derived from *in vitro* studies outside of a living organism. Although reductionists approaches are necessary as building blocks, these observations must begin to be placed back into a larger context. Below, I will further explore what is involved in the assembly and function of the NKIS in the context of ADCC and how we have amassed this knowledge.

## Shedding “Light” on NKIS Dynamics

The term “synapse” refers to a junction between cells and is most often used to describe the junction between neurons. This definition has since been expanded to also describe the junction between immune cells and TCs (71–73). While most notably used to describe the T cell IS (72), the term has more recently been expanded to NK cells (74). In both cases, the IS is a delicate ballet of receptors and ligands, cytoskeletal rearrangements and exchange of cytotoxic material in order to specifically destroy a cell deemed a threat. Understanding the players in this immunological dance and how they dynamically move through the process of immunological attack is vital to understanding ADCC and how antibodies affect the process and outcome.

The NKIS can be likened to a busy street corner at rush hour, people and cars crisscrossing and making their way to destinations in a concerted spatio-temporal fashion. Similarly, within the IS, receptors and cell surface molecules must bind to their ligands, signal and move to make way for the next set of molecules to follow suit. Understandably, evaluating the role of an antibody in this context can be extremely challenging. Do we use the detailed approaches of biochemistry and structural biology, medium resolution approaches provided by light microscopy, or more global “omics” types of big data acquisition and analysis? Ideally, details could be obtained equally from any one technique; however, technology has been historically limiting and complete understanding will require integration of all these techniques. In this section, I will provide a general overview of what is known to occur during formation of the NKIS, specifically during ADCC, and how structural biology and light microscopy have brought complementary understanding to this process.

### ADCC and the NKIS

**Figure 3:**
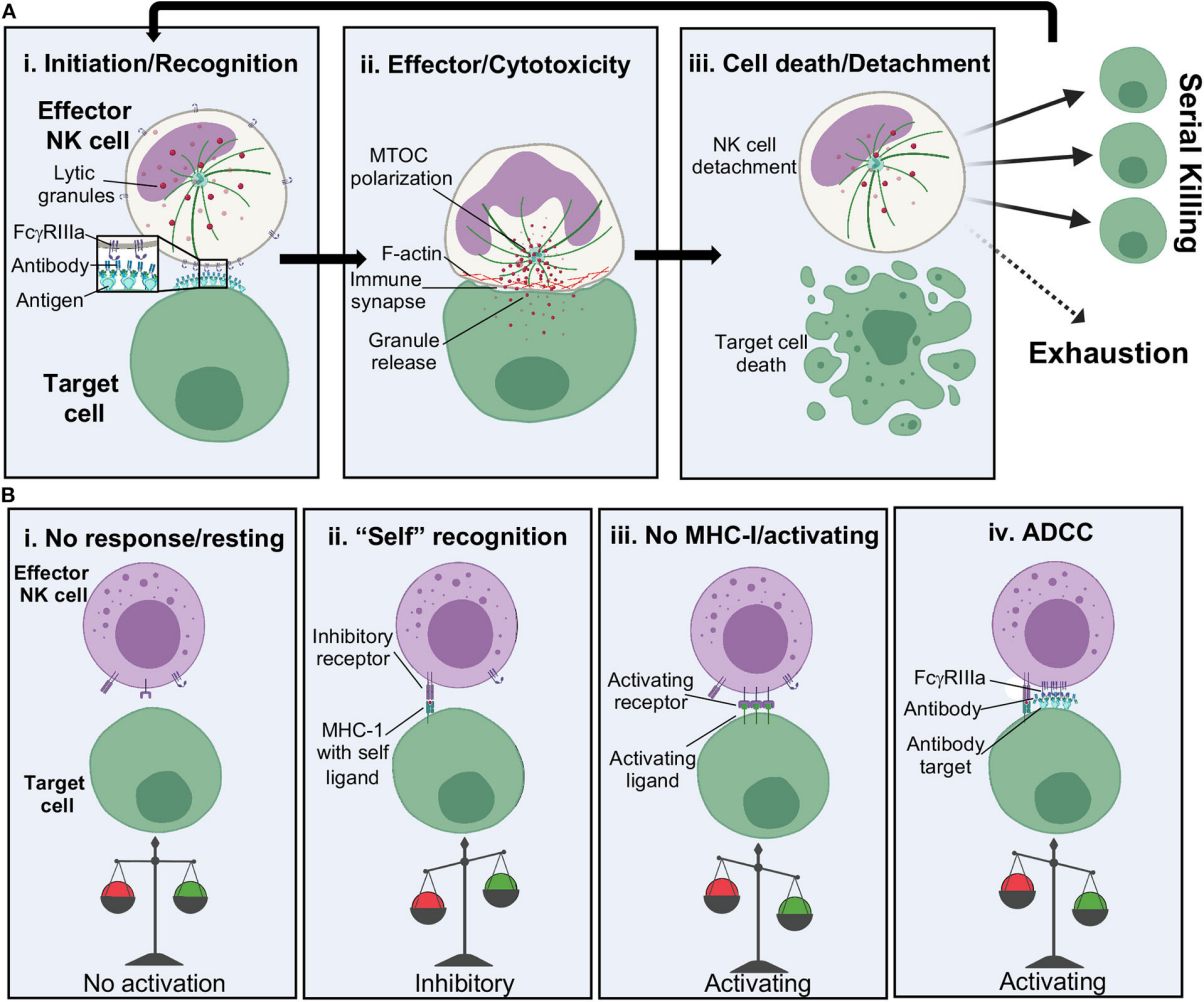
NK cell cytolytic activation. **(A)** Stages of NK cell cytolytic activity including: (i) tethering of effector to target cell and receptor binding, (ii) lytic granule and actin polarization at the immune synapse and release of lytic granules into target cells, and (iii) target cell death and release of the NK cell. NK cells can go on to serially kill or become exhausted. **(B)** NK cell activation relies upon a balance of activating and inhibitory signals. MHC, major histocompatibility complex.

The NKIS has been previously studied in detail and the general stages well laid out (Figure 3A) (74–77). There are several different types of NKISs (78), but we will focus mainly on the lytic IS here. In every type of synapse, the initial stages are the same, which is that of surveillance (Figure 3Ai). The process of surveillance involves the tethering of effector to target cell followed by adhesion. Each of these events is not completely well-understood, but likely involves carbohydrate sensing by CD2 (79), selectins like L-selectin (80) and integrins like CD11a/b and CD18 (77), which are upregulated and cluster early in the NKIS (77). These initial steps serve as way to lock effector and target cells together to then proceed to recognition, although some level of pre-activation occurs.

Following attachment to a target cell, it is time for the NK cell to decide: friend or foe? (Figure 3Ai) Since NK cells are primed to respond quickly and harshly to threats, their activation relies upon a well-controlled balance of activating and inhibitory signals (Figure 3B). Recognition of major histocompatibility complex I (MHC-I) bearing “self” peptides is an important part of this decision but can be overcome by stress signals. For example, certain cancers cause upregulation of stress signals such as MHC class I chain-related protein A/B (MICA and MICB) and UL16-binding (ULBP16) family proteins, which are recognized by the activating NK cell receptor natural-killer group 2, member D (NKG2D), leading to direct killing (81). Further, downregulation of MHC-I can occur during viral infection, also leading to direct killing of infected cells (82, 83). The presence of antibody coated cells can also lead to activation by ADCC. For ADCC to occur, surface expressed FcγRIIIa will recognize antibody bound to the surface of a TC (84), causing the formation of microclusters (85–87). This may be in part aided by concurrent cytoskeletal rearrangements, such as F-actin rearrangement, that is thought to aid in the clustering of receptors (76, 77, 88, 89) as well as the presence of lipid rafts to assist in fluidity (90, 91). Such rearrangements set the stage for microtububle polarization and delivery of lytic granules present throughout the NK cell cytoplasm to a conduit point (Figure 3Aii). Lytic granules bring CD107a to the openings in actin networks and are a tell-tale sign of NK cell activation and cytotoxicity (89, 92).

The release of perforin and granzymes at the synaptic cleft, the point of release of lytic granules, starts to signal the end of ADCC and cytotoxicity (Figure 3Aiii) (93). How the NK cell concludes cytotoxicity is still not well-understood, but proteolytic cleavage and shedding of FcγRIIIa ectodomains is thought to contribute to NK cell release (94). Although NK cells have been shown to serially kill multiple targets in a matter of hours, continued stimulation of ADCC via FcγRIIIa can exhaust the NK cell leading to decreased perforin release over time and a slower recovery of FcγRIIIa expression on the surface of NK cells (93–95). Serial killing can proceed until granzyme stores are out, leading to upregulation of CD95L, the ligand for target cell death receptors, resulting in slower apoptosis-mediated killing (96).

In addition to the formation and function of an IS, NK cells also release cytokines and chemokines that can exert effector activity on target cells and help recruit other effector cells, such as macrophages, dendritic cell and T cells as well as the proliferation of additional NK cells (97–99). Such cascades of activity all stem from the initial stages of antibody binding. Thus, elucidating the molecular basis of antibody-based activation of NK cells is fundamental to understanding the regulation of all downstream processes.

### Structural Biology as a Tool to Study the IS

Structural biology has been a key driver in our understanding of antibody interactions with both antigen and Fc-receptors. Crystallography has long dominated our understanding of Fab-antigen interactions. More recently, single particle cryo-EM has been increasingly important for determining antibody interactions, including more biologically relevant constructs of antigens and difficult targets. For example, cryo-EM is superbly suited to handle sample heterogeneity, enabling the structural analysis of diverse polyclonal antibody epitopes in a single imaging experiment. This technique has been instrumental in understanding the antibody-based immune response to viral infection as well as novel vaccines (100, 101).

**Figure 4:**
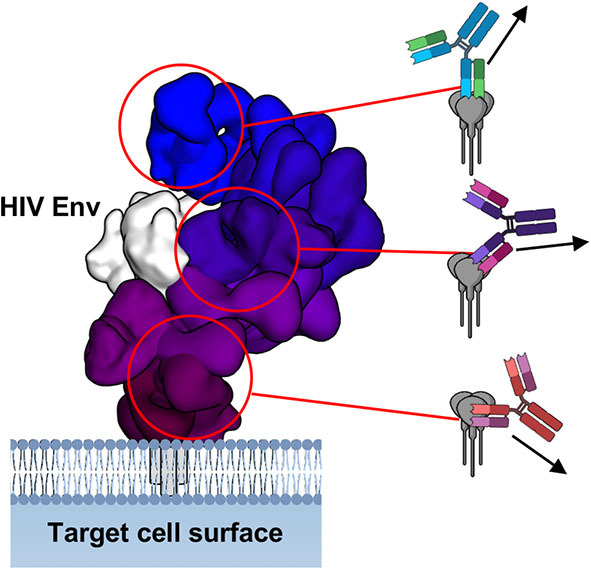
Fab-antigen structures give clues to antibody Fc presentation. Overlay of structures of Fab-HIV Env interaction, demonstrating a wide range of antibody angles-of-approach. On the right are schematics of how the full IgG would bind and the direction in which the Fc may point toward approaching effector cells. Figure adapted from Murin et al. (102).

In terms of epitope mapping, the field of infectious disease has exemplary examples of survivor-derived monoclonal antibodies bound to viral entry-associated proteins, which my colleagues and I recently reviewed (102). Such examples include, but are not limited to, HIV, influenza, ebolaviruses, marburgviruses, SARS, MERS, Hepatitis, Chikungunya virus, Zika virus, Dengue virus and Noroviruses, among many others. Antibodies are capable of binding to nearly any epitope presented on enveloped viral antigens (Figure 4), however their capacity to induce ADCC varies widely (103–105). The reason for such variance is unknown but may be related to where an epitope is located and the way in which an antibody binds, as well as genetic variation in FcγRs (106). The Fab alone bound to antigen can give clues to how the Fc may be situated and how this relates to receptor binding and macromolecular complex assembly (Figure 4).

By far the most important contributor to antibody ADCC activity studied so far is antibody subclass and glycosylation. While the basis of subclass remains somewhat of a mystery, we do have substantial evidence as to the importance of Fc glycosylation (43, 47, 49, 105, 107, 108). Specifically, if the glycan at Asn 297 is fucosylated, then the binding to FcγRIIIa is impaired (43, 47–49, 109). With removal of this core fucose, however, affinity is bolstered to the low nanomolar level. Further affinity can be gained from di-sialylated, complex glycans lacking core fucose, which also have strong anti-inflammatory properties (107, 108, 110). Nuclear magnetic resonance (NMR) studies suggest that the type of glycan attached to the Fc modulates Fc dynamics as well through the C'E loop that contains Asn 297 (111, 112). Conversely, FcγRIIIa glycosylation itself can also influence binding of IgG, hinging primarily upon a single glycan at Asn 162 (107, 108, 110, 113). Indeed, there are vast donor-specific differences in monocyte derived FcγR glycoforms that could influence the effectiveness of antibody therapies as well as donor-derived cell therapies (110). A more complete understanding of glycosylation effects on ADCC, as well as innovative ways to generate uniform and specific antibody glycosylation targeted for donor phenotypes, is an area of active research.

### Experimental Setup for Imaging the IS

Fluorescent light microscopy provides the unique advantage of being able to observe live cells, illuminating IS dynamics such as the spatial arrangement of receptors and ligands over time. Work in this area has been pioneered by the study of the T cell IS, which has been extensively reviewed elsewhere (7, 9, 76, 114–119). Study of the T cell IS has been in part driven by a more reductionist approach to parse the very complex IS into more digestible pieces. These techniques have now been adapted to study the NKIS as well.

The first obstacle to overcome when addressing the question of the role of antibodies within the IS is how to set up and observe single cell interactions. This is especially critical in ADCC since antibodies will influence the earliest stages of IS formation, and therefore timing is crucial. There are several technologies that have been developed to address this issue, but they fall into two major categories: imaging live cells enclosed in a physical space and imaging live effector cells interacting with a synthetic surface representing a TC (114). I will briefly touch on a few examples here.

**Figure 5:**
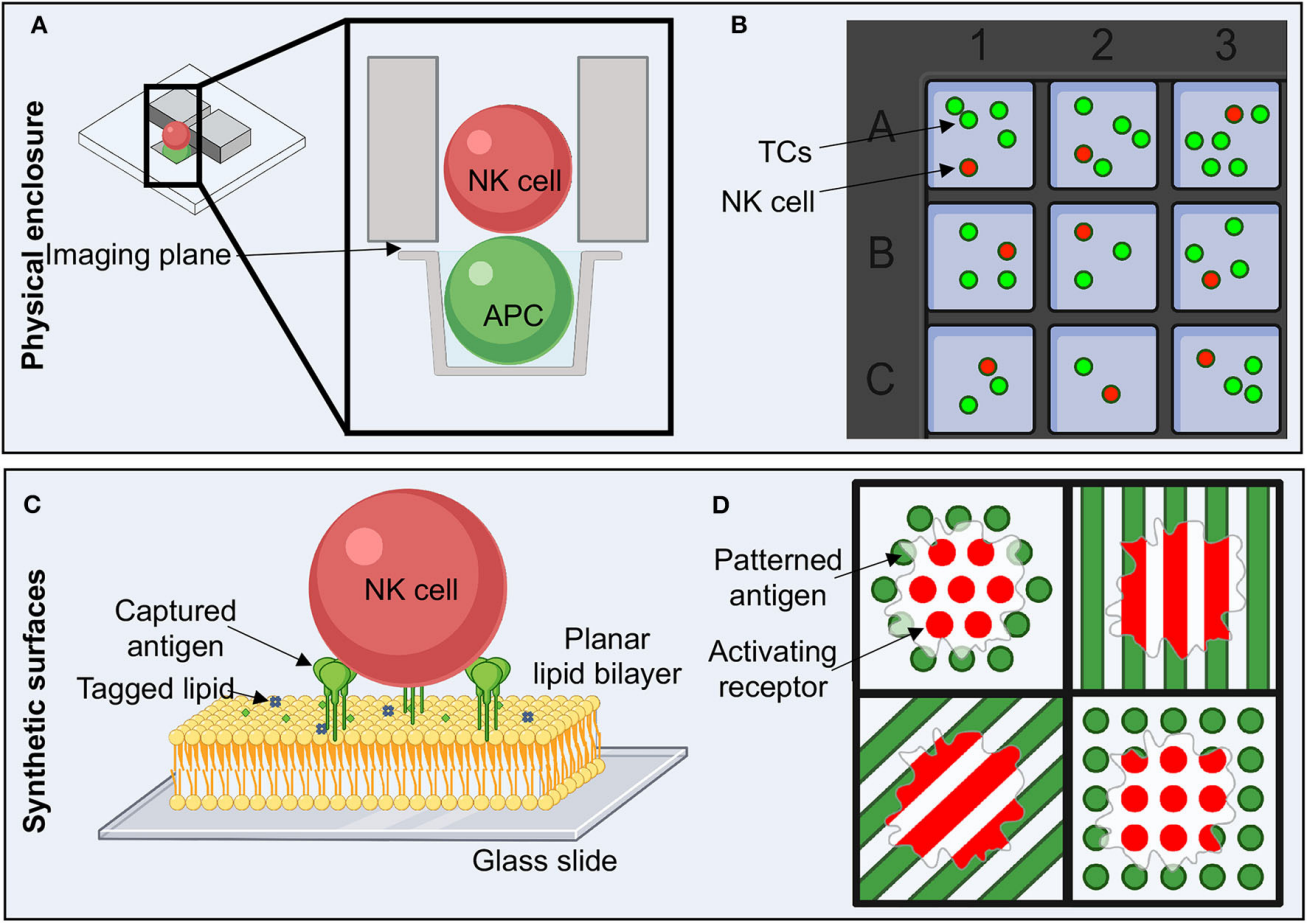
Experimental setup to study NK cell activation and the immune synapse. **(A)** Flow cells with microwells allow the physical isolation of single cell pairs to image the immune synapse in the z plane or *en face*. **(B)** Microwell plates enable the isolation of a small number of TCs and NK cells in order to study live, non-adherent cells. **(C)** The use of planar lipid bilayers on glass slides is a reductionist approach to studying the NK cell immune synapse. Antigens can be attached to chemically modified lipids, enabling ease of titrating antigen density and type as well as lipid composition. **(D)** Activating ligands can be pattered in order to determine how spatial and geometric constraints affect many aspects of NK cell activation. TC, target cell.

The first technique of imaging live cells requires physical isolation of these cells (Figures 5A,B). One approach for this has been the design of microfabricated wells, which are limited in diameter for single cells, but deep enough to allow a second cell to stack on top (120). This allows the imaging of the z-plane between the two cells where all the action of the IS takes place. Similarly, microfluidic chambers can trap pairs of cells and provide both face-on and side views of the IS (Figure 5A) (121). Both of these techniques have the advantage of high throughput but suffer from limits in imaging resolutions inherent to the microscopy techniques required for live cell imaging. Optical tweezers, which can capture an TC and present the IS to the focal plane of the microscope, also offer an intriguing solution for examining the NKIS in real time with improved resolution, however, without high throughput (122–124). This technique has been useful in predicting the effectiveness of chimeric antigen receptor (CAR)-modified T cells and may have similar usefulness for CAR-NK cells (75).

Cells can also be trapped within microchambers, which limits the range that non-adherent cells can move (Figure 5B) (125). This allows the free movement of effector cells in real time and facilitates tracking of single cell movement. With the additional implementation of acoustic signals, cell to cell interaction is stimulated, which allows for increased observations (126–128). Here we are not looking directly at the IS, but rather looking at whole cell behaviors within the context of a more “real” environment. One can envisage the grafting of tissues into these chambers to observe NK cell infiltration or to add different antibodies into media within chambers to observe the effects on whole cell dynamics within the context of ADCC.

A more reductionist approach to studying the NKIS utilizes synthetic forms of TC surfaces (Figures 5C,D). These systems are convenient for varying the type of antigen and also introducing spatial and geometric constraints. Supported lipid bilayers (SLBs) have gained traction in their utility to study the IS, as they allow for maintenance of the type of fluidity that would be encountered in cell membranes (Figure 5C) (85, 114, 129–134). There are many different ways in which to assemble SLBs, which have historically also found high utility for studying the electrophysiology of ion channels, pumps and transporters (131, 135, 136). For utility in biology, SLBs are typically formed by generating lipid micelles in solution and then depositing these onto ultra-clean glass slides (85, 137). Bilayers can integrate capture lipids, for example that contain nickel or streptavidin on their head groups, that can subsequently bind tagged antigens (Figure 5C) (137–139). In this scenario, antigen density can be titrated, or lipid composition can be easily adjusted.

Antigens or activating ligands can also be deposited directly onto substrate in predefined patterns using printing techniques (Figure 5D) (120, 140). This option allows for well-defined spatial constraints that can assist in probing how discrete patterns or geometries influence cellular activation and the organization of the IS, even within a single cell. For example, in an experiment where activating and inhibitory molecules are placed in distinct patterns, NK cell actin cytoskeletal rearrangement is more intense and patterned around activating signal patterns than inhibitory patterns (Figure 5D) (141). While this technique suffers from the static nature of the antigen presented, distances may be tightly controlled, and multiple different antigen-antibody complexes could potentially be examined simultaneously.

### Imaging the IS With Fluorescent Microscopy

Fluorescent microscopy imaging techniques can provide a range of temporal and spatial resolution (117, 142–144). While some techniques allow dynamic temporal resolution, such as the tracking of events in real time, these often suffer from physical constraints that do not allow high spatial resolution. Wide-field fluorescence microscopy (WFM) gains back spatial resolution from deconvolution methods that allow sharpening of signal post-image acquisition (142). Laser scanning confocal microscopy (LSCM) is also quite often utilized due to ease, but loses temporal resolution due to slow scanning speeds, which impede looking at fast events like those happening in the IS (121). Here, spinning-disk confocal microscopy (SDCM) allows for quicker acquisition (10 to 100-fold over LSCM) with lower photobleaching (117). Total internal fluorescence (TIRF) microscopy has limited z-axis resolution but is quite useful for analyzing the IS, which occurs in a narrow plane (145, 146). Two-photon fluorescence microscopy (TPFM) can complement TIRF by allowing similar resolution but with the ability to look at subcellular properties (117, 147).

Conversely, spatial resolution shines in the realm of super-resolution techniques (76, 85, 89, 115, 142, 148). This type of microscopy is not limited by the wavelength of light like the above examples. Stimulated emission depletion (STED) microscopy uses two lasers to activate and immediately deplete fluorophores, offering the ability to image smaller volumes (115, 149, 150). STED can technically be used for live cell imaging but is still slower than SDCM and has limitations with fluorophores. Single-molecule localization microscopy (SMLM) techniques (151), such as photoactivated localization microscopy (PALM) and stochastic optical reconstitution microscopy (STORM), utilize special photoactivatable probes that can indicate the single XYZ (more resolution in XY and less in Z) location of molecules (152–154). SMLM techniques can be used on live cells but are more practical with higher resolution in fixed samples. More complicated equipment as well as image analysis algorithms have been developed to offer insight into T cell activation in 3D within living cells (148). Lattice light sheet fluorescence microscopy (LLSFM), for example, offers the next generation for studying live cell IS events, with much faster Z slice image acquisition than SDCM along with super resolution (155, 156). However, LLSFM will have increased utility once cost and complexity both go down.

### Future Techniques for Understanding the IS

The future of imaging and understanding the NKIS will rely upon two major factors. One will be the marrying of high-resolution techniques, offered by electron microscopy for example, with those of resolution limited techniques, such as light microscopy. The other will be making new technologies more widely available to biologists, which is only a matter of time (157, 158). Such a renaissance has been seen in the field of electron microscopy with the advent of user-friendly microscopes and data analysis software (159). As far as the former, I will highlight a few exciting developments to keep an eye on in the coming years.

**Figure 6:**
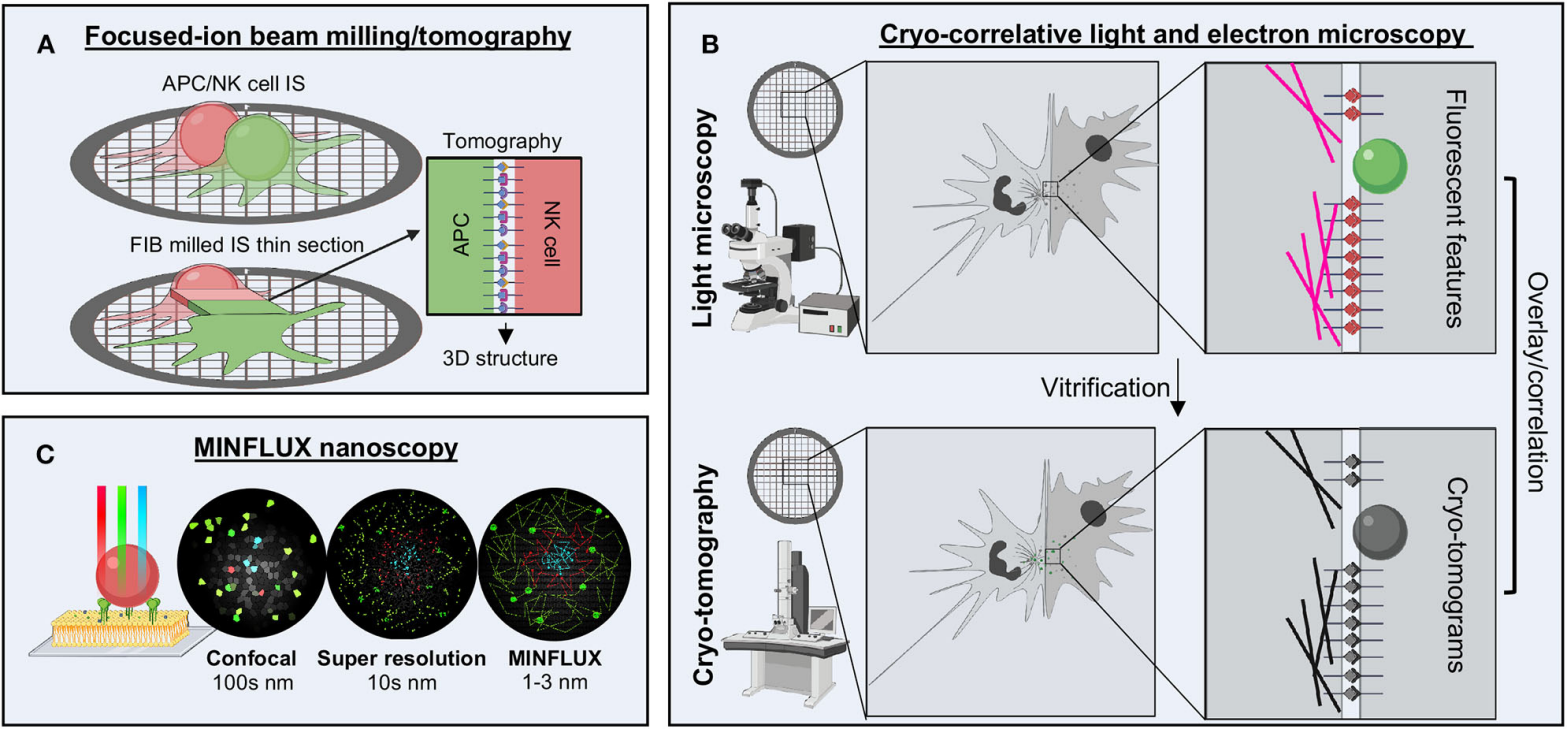
Future techniques for studying the NKIS. **(A)** FIB milling combined with tomography enables the generation of a thin layer to image the molecules bridged between cells. **(B)** Cryo-CLEM enables the localization of several structures within the immune synapse by super resolution microscopy followed by higher resolution structural analysis by tomography, which can be overlaid to provide additional details not offered by either technique alone. **(C)** MINFLUX nanoscopy offers the highest spatio-temporal resolution available of any light microscopy techniques, going beyond the limits of confocal and super resolution, down to the range of 1–3 nm. FIB, focused-ion beam; nm, nanometer.

In the realm of electron microscopy, the aspirational goal is to achieve sub-nanometer resolution of proteins and complex macromolecular systems *in situ*. Since most high-resolution techniques rely upon averaging, this is not readily possible, but more advanced techniques in cryo-electron tomography (cryo-ET) are quickly closing this gap (160–162). Phase plates, ideal for tomographic techniques to increase image contrast, have been instrumental in solving complex macromolecular assemblies within cells. Additionally, focused-ion beam (FIB) milling instrumentation allows for exquisitely thin and detailed cell sections to be isolated (Figure 6A) (163, 164). Cryo-ET examples include cytosolic and mitochondrial structures of actively translating ribosomes (165), complex actin and microtubule network assembly (166, 167), and intriguing views of the neural synapse (168). To examine the nuclear pore complex, detergent solubilization and removal of nucleic acids has enabled thinning samples as much as possible while maintaining 3D structure. Combined with integrative structural biology techniques, we now have the most detailed views of the intact nuclear pore complex ever seen (169). Cryo-ET, however, is labor intensive and requires a high level of expertise that has not become as streamline as single particle analysis. However, the field is rapidly moving toward automation and increased sophistication in data analysis of cryo-ET data. This field holds great promise as a tool for examining cell to cell contacts, such as the NKIS (Figure 6A).

Cryo-correlative light and electron microscopy, or cryo-CLEM, attempts to fill the gap between light and electron microscopy (Figure 6B) (170, 171). In this technique, whole cells are first imaged using fluorescent microscopy techniques to localize and identify features of interest. Next, cells are vitrified and imaged using cryo-ET, allowing for identification of features and subcellular location of the somewhat higher-resolution electron density maps generated. Genetically encoded fluorescent proteins allow the maintenance of cellular integrity and examination of fluorescence post-vitrification, or samples can be fixed, permeabilized and stained prior to freezing. Super resolution techniques are also starting to be combined to provide even more details (172–174). Cryo-CLEM may be a way to more accurately identify the location of signaling proteins within the IS and then extend results to the high-resolution context through electron microscopy.

Within the realm of light microscopy, a new technique has recently broken all the previous barriers associated with resolution limits, including spatial and temporal limitations as well as photobleaching effects. Known as MINFLUX, this revolutionary technique combines the super resolution techniques of PALM/STORM with those of STED by establishing the coordinates of proteins through minimal emission fluxes (Figure 6C) (175–177). This allows for nanoscale precision on the order of 1–3 nm spatial resolution. Moreover, this technique is adaptable to both scanning and standing-wave microscopes and can be used on fixed or live samples as well as in 3D. Tracking of single molecules within live *Escherichia coli* cells over long distances as well as highly detailed, multicolor labeling of the nuclear pore complex have been the earliest examples (175, 177). Clearly, this technique could be adaptable to tracking multiple different receptors within the NKIS. The only drawback at this current point is expense and availability.

## Geometric and Spatial Considerations Within the NKIS

IgGs are highly abundant within the human body at any given time, on the order of 7.5–22 mg/mL. Therefore, for ADCC to be an effective strategy for targeting cellular insults, NK cells must distinguish between free and specifically bound antibodies. This is thought to be achieved by the aggregation and agglutination that antibodies undergo upon antigen binding, whether to cell surface exposed antigens or soluble (178). This brings Fc domains in close proximity, allowing the clustering of cellular receptors. However, this explanation does not account for the sophisticated arrangements that signaling receptors must adopt in order to propagate a real signal, nor does it explain the reason and mechanisms associated with the variety of antibodies, receptors and glycoforms that exist. There is evidence to suggest that antibody arrangement is crucial for effector functions to proceed, that geometry and spacing can tune responses and that antibody allostery may also assist in regulating cellular activation. Below, I will discuss more detailed current knowledge of the early stages of antibody-based signaling and activation and provide examples that point to the concerted molecular underpinnings of effector functions.

### Initial Stages of ADCC

Once an NK cell has docked with a potential target, if opsonized antibodies are present, then FcγRIIIa will subsequently bind to those IgGs. Alone, the binding affinity of FcγRIIIa for IgG is estimated to be in the high nanomolar range (at least *in vitro*) but also depends on the genotype of individuals (31). For activation to proceed, however, the affinity between FcγRIIIa and IgG must be strong enough to allow for sustained interaction. IgG affinity is provided by the aggregation of IgG on immune complexes, increasing antibody avidity. Antibody aggregation is necessary because FcγRIIIa must adapt to a molecular arrangement that allows intracellular phosphorylation of cytoplasmic domains. Such an arrangement provides a platform for kinase binding and activity that is absent in monomeric FcγRIIIa. It stands to reason that there must be discreet forms of FcγRIIIa activation complexes beyond what simple aggregation implies. Indeed, there is evidence to suggest that such a form could be dimeric, as we will discuss more in the next section.

Once antibodies have successfully bound to the α subunit of FcγRIIIa through the ectodomain, this signal must be propagated to the intracellular side of the NK cell. This is achieved by co-stimulatory signal adapter molecules, which for FcγRIIIa is either FcεRI g or CD3 ζ (CD247) (Figure 1B) (13, 69, 70). These adapters were first attributed to the FcR for IgE (179, 180) and the TCR complex (181, 182), respectively, but are also adaptable to FcγRIIIa for ADCC. The γ or ζ adapters exist as a single pass transmembrane protein that forms a dimer through a cysteine bond (183–185). There appears to be no preference for either as they are found equally associated with FcγRIIIa (184). Together, the adapter homodimer and FcγRIIIa monomer are thought to form a non-covalent three-helix bundle (184). Mutations that dissociate adapter molecules from FcγRIIIa have been shown to prevent cell surface trafficking and are also thought to prevent FcγRIIIa degradation (184, 186).

### ADCC Signaling

Although the overall structural motif of the macromolecular signaling complex has yet to be elucidated, once FcγRIIIa self-associates, downstream signaling can then proceed. The γ or ζ activating adapter molecules contain cytoplasmic tails with ITAMs (187). In the proper conformation, these ITAMs can be phosphorylated at two of 6–8 tyrosine sites, setting up a docking site for Src-family kinases. It may be possible that Src kinases dock and rely on a specific dimeric motif of associated FcγRIIIa and adapters for the kinases to dimerize themselves and auto phosphorylate, structurally similar to what has been shown for the JAK2 kinases (188). Indeed, Src dimerization is predicted to be necessary as its role as a hub for multiple signaling activities (189–191).

Once phosphorylated, the signaling adapters are ready for recognition by Syk or Zap70, for example, through tandem SH2 domains (192–194). Syk or Zap70 interaction with phosphorylated ITAM domains leads to the downstream activation of several signaling pathways. Concurrently, FcγRIIIa cross-linking activates PLC-gamma enzymes to generate inositol 1,4,5-trisphosphate (IP3) and *sn*-1,2-diacylglycerol (DAG), leading to Ca^2+^ release from stores within NK cells, which is required for granule release. FcγRIIIa cross-linking also activates PI-3 kinase, which produces additional signaling molecules to assist in ADCC-associated activation activities. Additional associated signaling pathways include the Ras, ERK2, MAPK, Vav/Rac, and NFAT pathways. Each of these pathways leads to activities such as actin reorganization, cellular proliferation and cytotoxicity. Further, the JAK/STAT pathway can also be secondarily activated, leading to upregulation of cytokines and chemokines, recruiting other cells or enhancing effector functions (195).

Signaling is a highly complex and multicomponent process that can change depending on extracellular stimuli. For example, antibody activation via FcγRIIIa sets up ADCC with a particular response, but that response differs from direct cytotoxicity or activation inhibition (78, 196). Increased understanding of the complex signals that occur during NK cell activation will help us to understand how to modulate ADCC activity, perhaps through new designs of antibodies or a synergistic combination of antibody and small molecule.

### Receptor Movement and Lipid Composition

The initial stages of ADCC as well as the formation of the NKIS are both intrinsically linked to composition of the cell plasma membrane. Here is where membrane bound receptors interact both extra- and intracellularly to generate a robust reaction on the cellular level. Once thought to be a somewhat homogeneous environment, the cellular membrane is actually a circus of different elements, composed of a wide range of lipids that tightly control many cellular activities, including immune signaling. While the evidence for how lipid composition of cells is organized and influences cellular activities is not wholly realized, due to the difficulties associated with studying lipid composition *in situ*, there is still some compelling data that warrants discussion, especially in relation to immune signaling and ADCC.

The composition of eukaryotic plasma membranes is primarily of glycerophospholipids, with head groups attached to at least one unsaturated acyl chain (197, 198). While these lipids are sufficient to form a bilayer, it is known that sterols and sphingolipids also make up a large portion of the plasma membrane. The sphingolipids can be further classified into ceramide-based sphingomyelin or carbohydrate-based glycosphingolipids, both which are often saturated in their acyl chains. Of the sterols, cholesterol is the principle component. Both of these additional lipids are of much lower abundance in internal membranes but are made in the ER and Golgi and transferred to the cell surface. The composition of the inner and outer leaflets of the plasma membrane is also known to be different, with cholesterols and sphingolipids thought to preferentially reside in the outer leaflet.

More than any other component, cholesterol changes the properties of the bilayer by increasing rigidity and reducing permeability, while still allowing free-lateral movement of proteins and lipids. Model lipid studies indicate that cholesterols and sphingolipids form distinct domains within the more fluid background of the plasma membrane, often referred to as lipid rafts, and that transmembrane proteins can be included or excluded from these domains based on their own physical properties (199–202). Within the outer leaflet, GPI-anchored proteins are enriched (203). These are subsequently linked to the inner leaflet by signaling proteins that are preferentially found in this portion of the plasma membrane, forming signaling platforms.

Evidence for such signaling platforms, especially in regard to FcγRIIIa as well as its associated signaling domains, is compelling in NK cells (90, 204–208). Immunoregulatory elements can be preferentially partitioned within different lipid environments, with positive signaling components such as the Src and Lck family kinases being found in cholesterol rich lipid rafts, while negative regulators such as phosphatases are excluded from these regions. By clustering these elements within microdomains, signaling can be more easily and readily achieved. Although NK cell ADCC is not associated with GPI-anchored proteins, many activating immune receptors are indeed GPI-anchored, which also suggests that immune signaling is biased toward lipid rafts.

**Figure 7:**
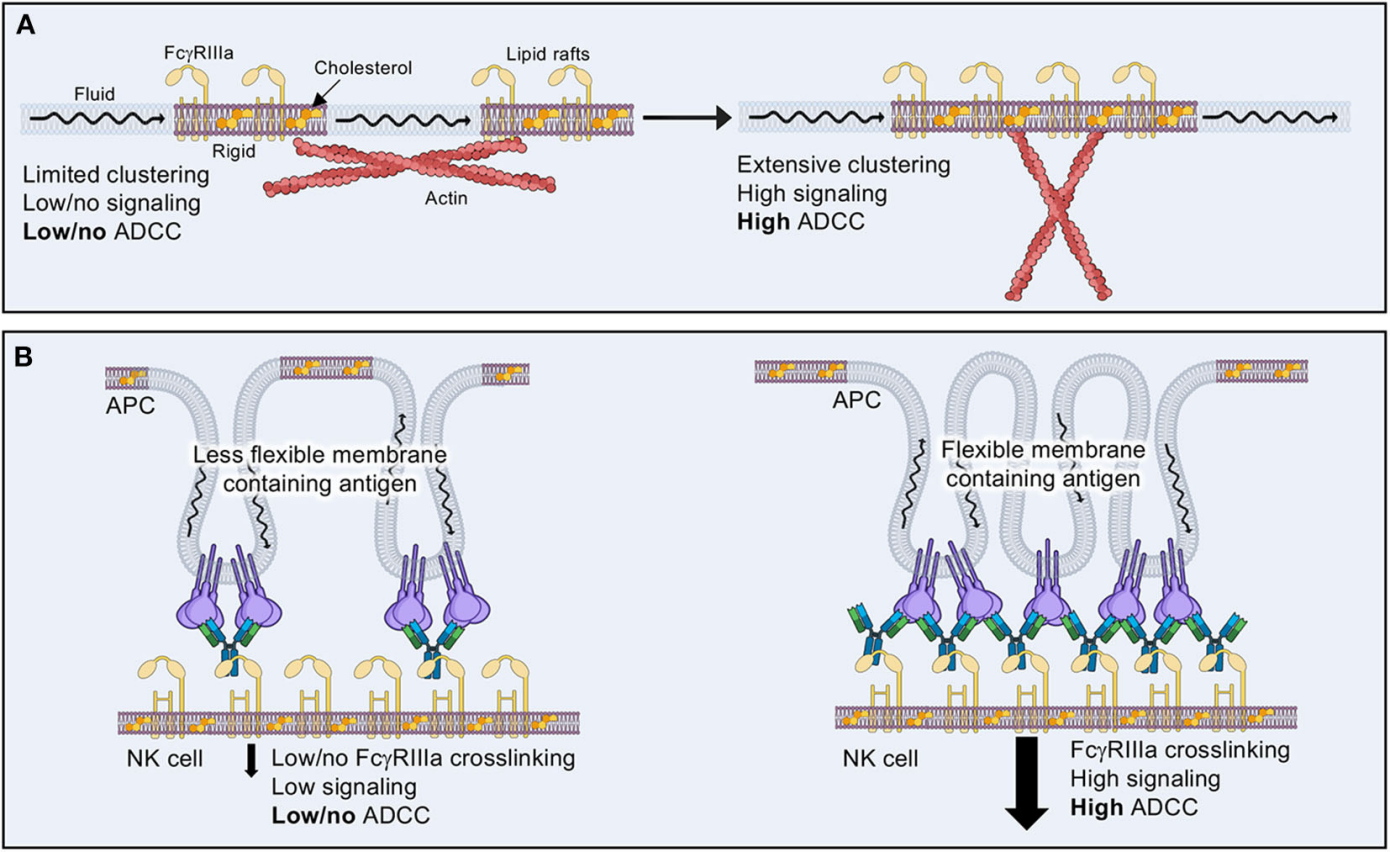
The effects of lipid composition on ADCC. **(A)** Lipid rafts containing activating receptors and higher levels of cholesterol tend to be more rigid and isolated in the plasma membrane of resting or inactive NK cells. Actin cytoskeletal rearrangements are thought to aid in bringing lipid rafts together in order to allow tighter clustering of signaling molecules and increased cytolytic activity. **(B)** Target cell membrane lipid composition may aid or inhibit NK cell ADCC by how well antibody coated antigen is able to cluster, thus promoting FcγRIIIa receptor clustering.

Evidence suggests that negative regulation of NK cell cytotoxicity results from blocking the association of activating receptors within lipid rafts (Figure 7A) (204, 205, 207). It is thought that actin cytoskeletal rearrangement assists in the association of lipid rafts containing positive regulatory components within the immune synapse (208). If negative regulatory components dominate the signaling platform, then actin rearrangements can be blocked, thus limiting the rearrangements of downstream signaling components to lipid rafts containing primarily positive signaling elements. This may explain why actin cytoskeletal rearrangement is one of the earliest and fastest physiological responses to NK cell activation.

Lipid rafts likely alter the way in which signaling components interact once liganded to a target cell. When considering the many ways in which an antibody bound to its target antigen could be presented to FcγRIIIa, it is important to consider how such geometric and physical constraints may be affected by the more rigid confines of a lipid rafts. Indeed, cholesterol enrichment seems to be a harbinger for more efficient NK cell cytotoxicity (209), but there is no evidence to address how this affects ADCC or how the physical arrangement of antibodies within a macromolecular signaling complex may influence activation. Conversely, the lipid environment of a target cell could also affect how antibody-bound antigens are presented within the IS (Figure 7B) (210). Much less attention has been paid to the target cell side of NK cell cytotoxicity; however, some evidence suggests that lipid composition is vital to the sensitivity of target cells to attack (210). There is much room for exploration in the realm of lipid composition and its influence on ADCC, which may in turn have important implications for the choice of antibody used for immunotherapeutic purposes.

### Spatial and Geometric Constraints Within the IS

For NK cell signaling to occur, extracellular signals must be propagated across the cellular membrane. This necessitates some type of unique arrangement of proteins that differentiates a resting cell from a cell that is detecting something in the extracellular environment. In the case of ADCC, this starts with understanding the arrangement of FcγRIIIa. Is there a singular structural motif that must be achieved in order for activation to occur? Or is the arrangement of these receptors more stochastic and tunable to the subtleties defined by extracellular factors? Like most realities of biology, the answer likely lies somewhere in the middle.

In an analogous system, the T cell immune synapse, much work has already been done to answer these questions, ultimately setting a paradigm for lymphocyte-based signaling (181, 183, 185, 211–215). Similar to FcγRIIIa, the T cell receptor (TCR) is composed of extracellular domains that recognize peptide bound MHC-I. These domains, a heterodimer of α and β domains, are single pass transmembrane proteins that must also pair with adapters to propagate signal. Nucleation is accomplished with the CD3 hexamer, comprised of heterodimers of CD3 γε and CD3 δε as well as CD3 ζζ, which is one of the same adapters used by FcγRIIIa. The resultant supramolecular complex is thought to be the basal unit for signaling and requires very tight spatial interaction, which was recently resolved by cryo-EM, revealing a crisscrossing network of transmembrane subunits (185). Ligand binding does not induce any obvious structural changes, with the caveat that this structure utilized glutaraldehyde fixation. There are some single molecule data as well and NMR studies that suggest that reorganization within the TCR signaling complex may occur upon ligand binding still (216, 217). Previous structural data also suggested that a complete signaling complex in solution may be dimeric (211). It is thought that the antigen-bound TCR then interacts with actin and other signals to function as a mechanosensory unit (123, 218).

Studying microcluster formation and dynamics of signaling kinases that are anchored to the plasma membrane has been critical to our understanding of TCR signaling and provides many lessons for studying the NKIS. Studies using PALM revealed that in cells activated on glass coverslips, associated signaling molecules like LAT and SLP-76, which links to actin filaments, form in much smaller nanoscale sized clusters than previously postulated (146, 219, 220). The increased spatial resolution of PALM also revealed that signaling could occur in nanoclusters that may only contain signaling units as small as dimers of the TCR as the minimum requirement for signaling (221–224). Complementary studies using light sheet STORM of activated T cells from mice showed similar types of spatial organization occurs *in vivo* (225).

On the antigen side of immune activation, antigen presentation and spacing seems to be critical for thresholding T cell activation. Several studies using nanoscale spacing of activating molecules suggest that differential activation can be achieved depending on the space provided within the IS (114, 212, 226, 227). One study integrating both lateral and axial spacing of antigen determined that tight 2D clusters with limited axial spacing of <50 nm was an ideal arrangement for T cell activation (212). Such spacing is thought to fortify clustering based activation while excluding CD45, which must exit the IS in order for activation to proceed. Another study concluded individual activated TCRs may contribute more to T cell activation than overall clustering (223). This adds some clout to the idea that there is a necessary arrangement of the TCR that qualifies activation, potentially a dimer as previously suggested.

**Figure 8:**
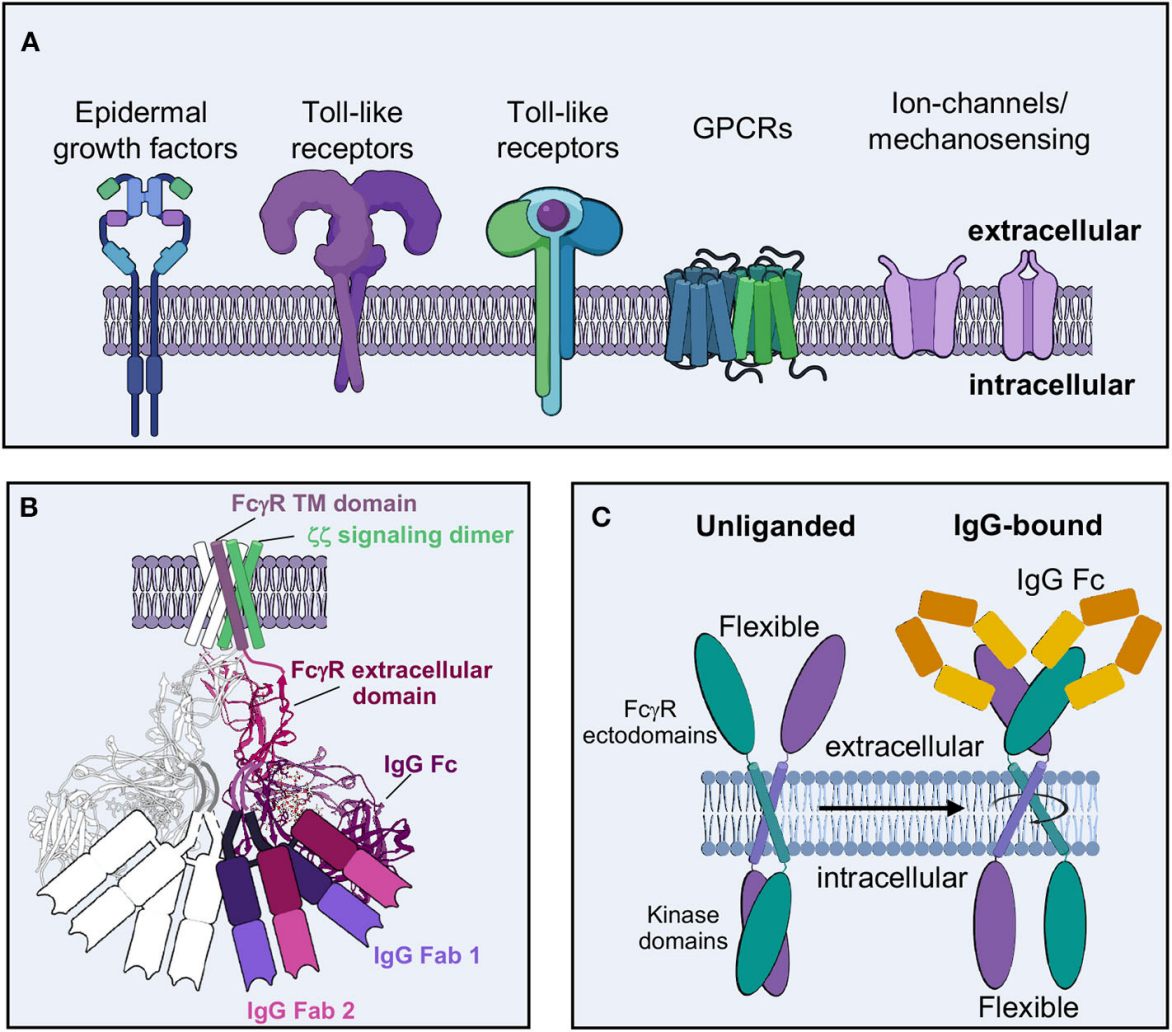
Molecular basis of effector cell activation. **(A)** Dimers are prevalent molecular motifs in cellular activation throughout the immune system. **(B)** Potential dimer model of IgG-FcγR-CD3 ζζ activation complex, based off structures of complex components [FcγRIIb-Fc from PDB-3RY6 overlaid on FcγRIIb crystal contact dimers from PDB-3RY5; model of transmembrane assembly of CD3 ζζ structure from PDB-2HAC and model of FcγRIIIa/adapter assembly from Blazquez-Moreno et al. (184)]. **(C)** Potential model of downstream antibody-mediated signaling based on the “rotation model,” which postulates that intracellular kinase domains are exposed for phosphorylation upon proper dimer assembly when extracellular activating ligands bind.

For ADCC-based activation in NK cells, there is evidence to support a signaling complex may involve FcγRIIIa dimers, which have greater appreciable binding to IgGs (14, 70, 184, 228). Dimers are quite prevalent throughout signaling biology and are thought to generate universal platforms for kinases with broad activity (Figure 8A) (181, 191, 213, 229–232). Cytokine mediated signaling provides the richest examples of dimer mediated signaling, with a large diversity of structures induced by cytokine binding (229, 230, 233). There are many additional examples of homo- and heterodimeric complexes that drive signaling, including growth factor receptors, insulin and other hormone signaling receptors and nuclear receptors (Figure 8A). In each of these cases, dimerization may occur in several different stoichiometries and can orient dimers in a plethora of ways. Further, toll-like receptors also require extracellular antigen-based dimerization for signaling to occur (Figure 8A) (234). Given the diversity of dimerization in signaling, it seems highly likely that ADCC signaling may follow a similar type of organization.

The idea of signaling dimers in ADCC has been previously postulated. Artificial dimers of FcγRIIIa were sufficient to reproduce NK cell activity and conversely increase affinity for IgG (228). Additionally, ectodomains of FcγRIIa within a crystal lattice suggest a possible domain arrangement that may extend to other FcγRs. Mutation of critical residues in this dimeric interface demonstrated reduced cellular activation but not ligand binding (235). Later structures of FcγRIIa demonstrated a new dimer interface potentially that serves as an activating arrangement of signaling as it could reasonably accommodate two opposing Fc domains as well as ligand bound Fabs (Figure 8B) (236). The authors proposed a model where constitutive dimers exist on the cell surface in an inactive arrangement that changes upon ligand binding, posing the intracellular signaling domains in an active formation. Perhaps this is similar to the “rotation model” of other signaling motifs, where less flexible intracellular domains are opened up for phosphorylation upon extracellular ligand binding (Figure 8C) (233). FcγRIIa, unlike the other FcγRs, does not require adapters for signaling, having its own cytoplasmic ITAMs. It is not clear if FcγRIIIa or other FcγRs exist as a similar constitutive dimer on the cell surface. Achieving a similar dimeric arrangement of other FcγRs ectodomains may be a critical component for successful ADCC and additional effector activities, as I will discuss in more detail below.

There is evidence for other types of dimeric signaling motifs that suggests that the immune response is not “on or off” in these structural motifs but rather may be tunable. For example, the cytokine Erythropoietin (EPO) and its associated receptor EpoR can be changed in their association topology by diabodies that re-orient their geometry and lead to differences in intracellular signaling (230). Other examples, such as tumor necrosis factor (TNF) signaling as well as some prokaryotic chemoreceptors such as Tar, further demonstrate that dimer formation alone is not always sufficient for signaling and that conformational changes may be additionally necessary (68, 232). Throughout all these examples, there seem to be thresholds and variations in geometries that lead to the idea that cellular activation can be tuned in almost any type of signaling event, possibly even NK cell ADCC.

### Complement Dependent Cytotoxicity and Comparison to ADCC

Complement is thought to be a more ancient form of immunity (237); therefore, principles of complement, especially for IgGs, may have important implications for ADCC as well. Complement is another function of the modular antibody that leads to target cell death without the need for effector cell activity. In complement, antibodies binding to cell surface antigen set the stage for additional complement associated proteins to assemble an activation platform ultimately leading to complement deposition and effector cell phagocytosis or assembly of the membrane attack complex (MAC) (238). A plethora of studies have previously set the molecular requirements for complement assembly, along with many isolated structures of complement related proteins, but it was tomography that really started to reconcile these observations (239–241).

**Figure 9:**
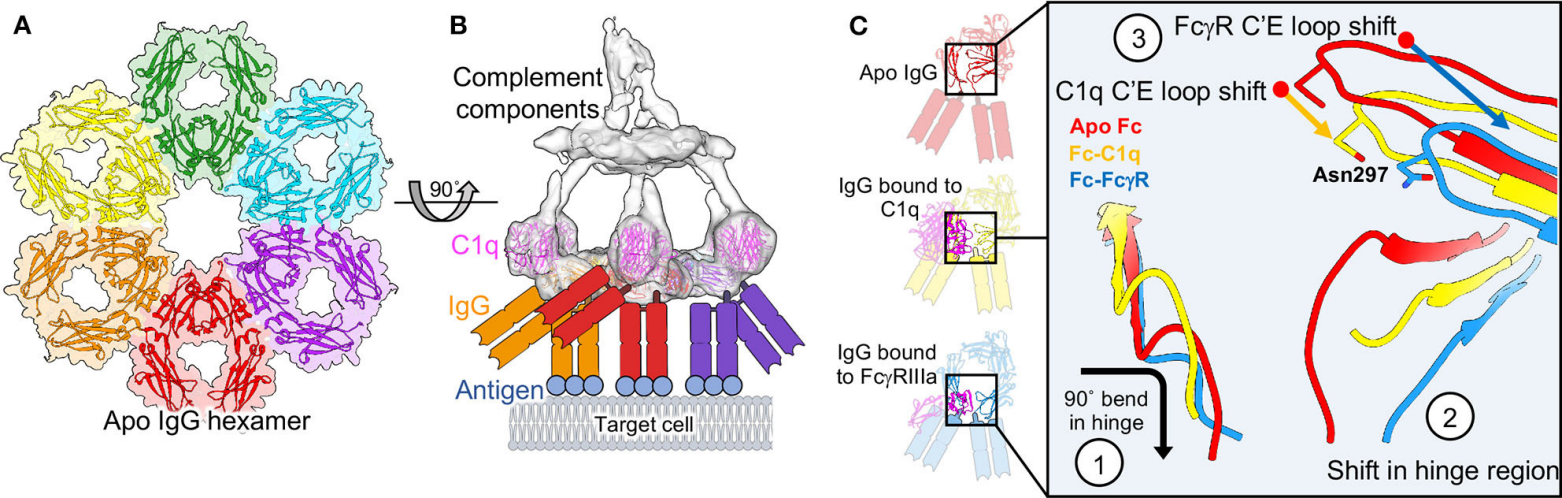
Molecular basis of complement and comparison to ADCC. **(A)** Fc hexamers form the basis for complement deposition (PDB-1HZH). **(B)** Tomography of complement deposition indicates that IgG Fc domains bend parallel to the antigen presenting cell, presenting the binding domain for C1q (EMD-4232, PDB-6FCZ). **(C)** Both C1q and FcγR binding induce similar conformational changes in the Fc domain compared to apo-Fc including (1) a 90 degree bend in the hinge-region loops, (2) a shift in the position of the hinge-region loops and a widening of the base of the Fc and (3) a shift in the position of the C'E loop, including the position of Asn 297 and the associated glycan (Apo Fc from PDB-1HZH, Fc-C1q from PDB-6FCZ and Fc-FcγR/FcγRIIIa from PDB-3WN5).

Revisiting a fortuitous observation of the first full length structure of a human antibody (242), Diebolder and colleagues tried to understand possible functional consequences of Fc interactions seen in crystal packing, seeding hexameric arrays (Figure 9A) (239). These hexamers were reminiscent of evidence indicating that IgG would need to form these types of structures in order for the complement cascade to proceed (243). Mutations that limited Fc-Fc interaction lead to decreases in complement activity while supporting mutations increased activity. Tomographic structures of assembled IgG on antigen presenting liposomes displayed the same type of hexamers seen in crystal packing (Figure 9B). Further, it was observed that Fc domains bend into a plane parallel with the antigen presenting plane, presenting the epitope for the first complement protein C1q, which was also observed in their structures when assemblies were made in the presence of complement proteins (Figures 9B, C). Interestingly, only a single Fab of IgG was necessary for complement deposition, freeing up the rest of the IgG structure to assemble properly (Figure 9B). Bispecific antibodies actually performed better in complement assays.

Later EM observations of pentameric and hexameric IgM also showed very similar structural assembly for complement, with many additional proteins and observations added on (240, 241). In these observations, both Fab arms of the IgM were bound to antigen, which suggests that either IgM and IgG antigen recognition geometries are different, or there is a dependence on antigen type. Previous studies on IgM alone indicated that these antibodies exist in a pre-bent shape that may help to influence complement binding, whereas IgGs tend to be much more flexible in solution (244–246). More recent high-resolution cryo-EM structures of IgM as well as IgA are beginning to give us even more detailed insight into how full-length antibodies operate pre- and post-receptor binding, hopefully providing lessons that can extend to other immunoglobulins (22, 247).

A recent structure of Rituxumab Fab bound to CD20 indicated that the antibody recognizes CD20 dimers and that Fab-Fab interactions are important to antibody binding (248). The authors presented some evidence that these types of Fab-Fab interactions may promote hexameric assemblies important for complement deposition. Fab-Fab and/or Fc-Fc interactions may be a much more prevalent antibody adaptation as there are more examples in the literature, such as the Fab-Fab interactions for some malaria antibodies (249).

Most intriguing is the folded presentation of Fc in both IgG and IgM complement assemblies, that is similar to those seen in Fc-FcγR structures (Figure 9C) (42, 239). Although FcγRs can bind free IgG, perhaps a folded Fc domain is required for proper FcγR dimer assembly and signaling. Varying degrees of Fc presentation based on antibody binding angle or the number antigens engaged by an IgG simultaneously may modulate FcγR dimerization, thus tuning the immune response and leading to the differential innate activities observed by antibodies with identical phenotypes but varying levels of ADCC.

### Antibody Allostery

**Figure 10:**
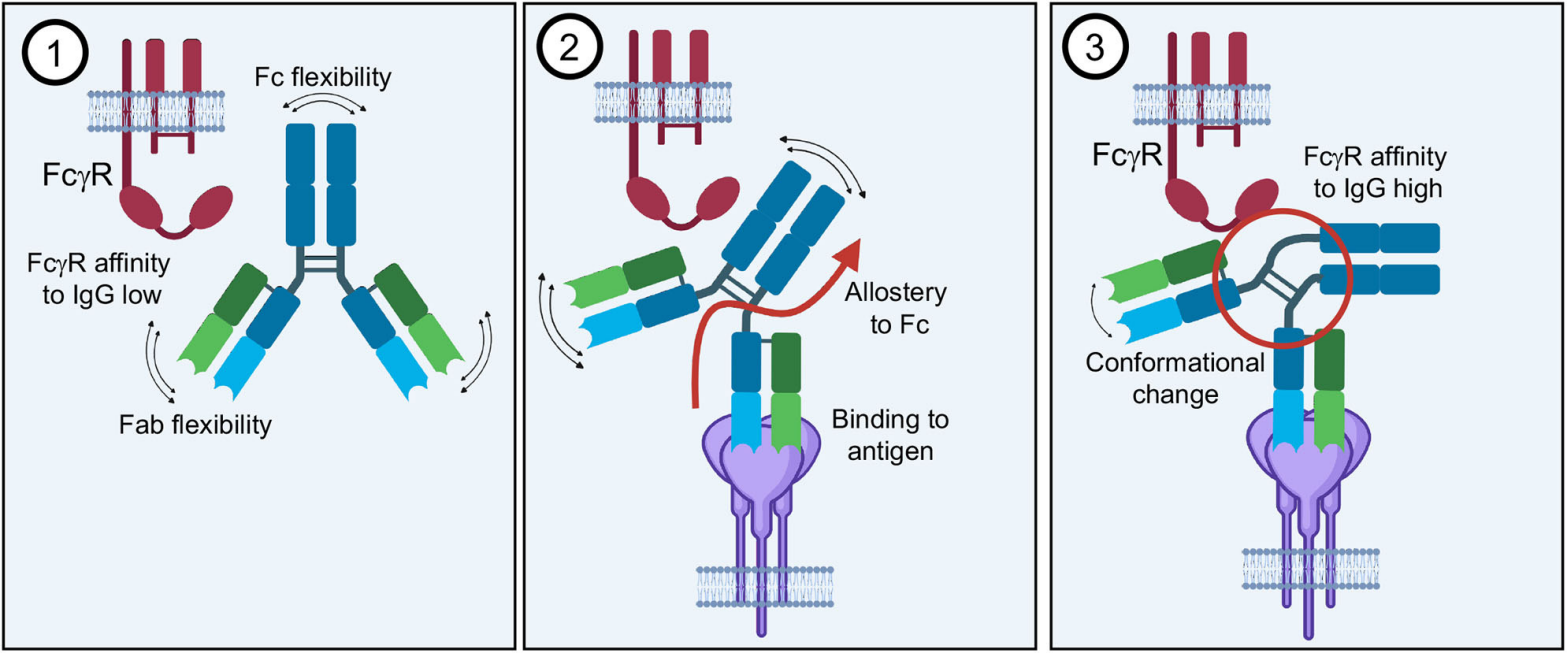
Antibody allostery. The theory of antibody allostery or “intramolecular signaling” asserts that (1) an apo-IgG has flexibility that impedes its affinity to FcγRs. (2) Antigen binding generates an allosteric signal from the Fab to Fc domain. (3) Allostery causes a conformational change in the Fc as well as decreased flexibility that facilitates FcγR binding with higher affinity.

The idea of antibody allostery or “intramolecular signaling” has been discussed for many decades and remains a debated topic in the antibody field (Figure 10) (250–253). This hypothesis proports that the Fab and Fc domains of an antibody communicate through structural properties inherent to antibodies. To that end, antigen binding in the variable domains would change structural conformations of constant regions, priming them for ideal FcR binding that would be lacking in the apo form of the antibody. However, the reigning theory behind antibody structure-function relationships is that Fab and Fc operate independently of each other, with the former binding to antigen and the later binding to FcRs without one affecting the other directly (178). Antigen-induced aggregation of IgG is thought to increase the relatively low affinity of receptor binding affinity through avidity and induce receptor aggregation that is required for downstream signaling. Without antigen, the 1:1 interaction of IgG and FcγR remains weak and/or does not induce receptor clustering even in high affinity interactions like FcγRI. While there is an ample amount of evidence to support this type of associative cooperativity, there continue to be models and complementary evidence that antibody allostery may play a subtle but significant role in Fc-mediated effector functions.

Studies of antibodies in solution demonstrate that there is a large degree of flexibility associated with the antibody structure, mostly likely imparting a wider sampling space for a diversity of target antigens (Figure 10) (244, 245). Furthermore, crystal structures of intact IgGs also indicate that the hinge-regions are largely responsible for this flexibility, although these regions tend to be poorly resolved and in the confines of a crystal lattice (68). However, detailed dynamics studies of antibody behavior in solution provide computational evidence that antigen binding changes the sampling space of Fc as well as the flexibility, providing a type of “rigidity” that is not found in unbound antibodies, which may lead to greater FcγR affinity in the presence of antigen (254).

There is some direct evidence to suggest that Fab binding also has consequences for the constant region of an antibody, at least within in the Fab domain. Comparison of several crystal structures of native and liganded Fab demonstrate changes that occur within the Fab hinge as well as elsewhere in structures (255). Although earlier studies comparing bound and unbound Fab run contrary to these observations, it appears that there are some antigen/antibody specific properties that may account for variability. This may also point to an explanation for differing innate properties of antibodies depending on the epitope.

Moving toward the Fc end of the antibody, there are several studies that indicate that perturbations to the Fc region can affect binding to antigen, for example with Pertuzumab (as an IgA1 and IgA2) and Her2 binding (256). Similarly, many other studies have shown that using identical variable regions with different constant regions (i.e., isotype or subclass switching) leads to variable antigen binding as well (257). Further, an antibody's ability to neutralize viruses is possibly linked to its ability to bind to FcγR and perform effector functions (258). Although indirectly, the above evidence suggests that Fc and Fab are intrinsically linked.

Looking back to complement, we now know that antigen binding is indeed required for the formation of IgG hexamers, leading to a bending of Fc and Fc-Fc interactions (239). Since these hexamers do not form stochastically in solution, and since membrane bound antigens likely do not stochastically form the hexameric shape required for complement deposition, a system of allostery seems a likely explanation for how antigen binding leads to complement associated macromolecular assembly (259). Higher resolution studies of the complement activation complex, perhaps using single particle studies, may be required to confirm this and to piece together what intermediate IgGs look like moving into the complement-bound state (259, 260).

Detailed structures of full IgGs alone will also provide critical data showing the structure of Fc in solution when not influenced by neighbors within a crystal lattice. Indeed, comparing structures of free Fc and Fc bound to FcγR show that FcγR binding induces a change in Fc from symmetric to asymmetric, which precludes a second FcγR from binding (Figures 1D, 9C) (12). It would be most intriguing to determine if antigen binding pushes Fc toward a more asymmetric shape, perhaps facilitating FcγR binding. The increasing capacity of cryo-EM to solve structures of very small, Fc-sized molecules as well as to deal with the type of sample heterogeneity associated with IgGs will prove to be a valuable tool toward these efforts.

Together, these data collectively fit into a model of leukocyte variability in relation to effector functions and suggest a mechanism that inherently must include allostery. This is not to suggest that antigen induced aggregation is not necessary, but that there are likely complementary mechanisms that lead to effector functions.

### Reconciling Antibody-Antigen and Antibody-FcγR Structures

**Figure 11:**
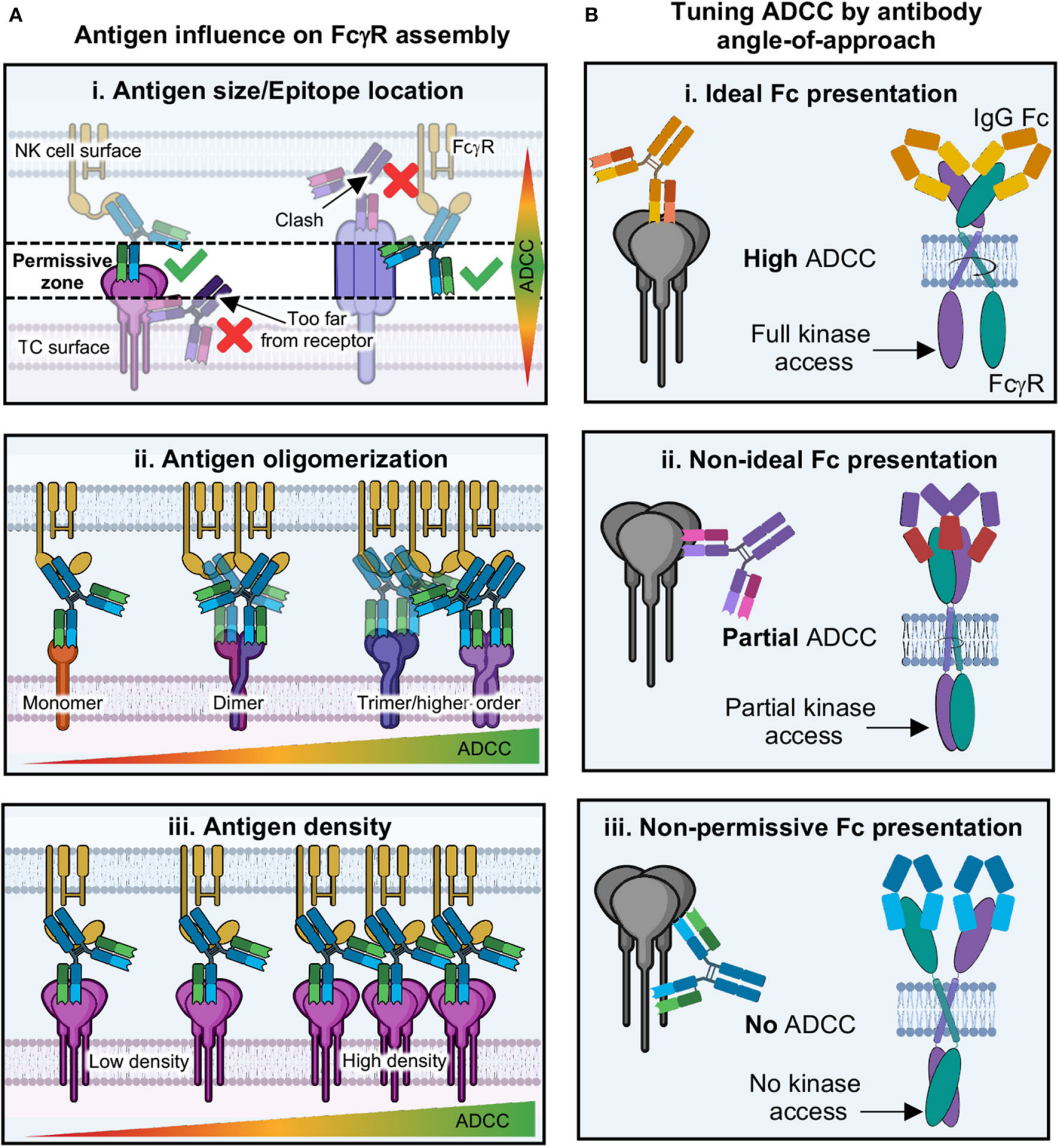
Proposed model of antibody tuning ADCC through activation complex geometry. **(A)** The immune synapse involves a finite distance between target and effector cell; therefore, the spacing requirements present a “permissive zone” in which antibodies can bind. (i) antigens that are short (i.e., Ebola GP) require antibodies to bind to epitopes on the top of the antigen, while antigens that are longer (i.e., influenza HA), require antibodies to bind lower down on the antigen in order for proper binding to FcγRs. (ii) Antigens that are monomeric may be less likely to facilitate FcγR dimerization/aggregation, while those with higher oligomers present multiple epitopes that increase avidity and chances for FcγR clustering. (iii) Antigens that are of low density may prevent ADCC due to the inability for enough FcγRs to cluster. Conversely, those with higher antigen density present more opportunities for antibodies to induce FcγR clustering and thus higher levels of ADCC. **(B)** (i) If antibodies bind to antigen in an epitope that presents Fc domains in a way that allows FcγRs to dimerize in an ideal way, then intracellular domains can be properly phosphorylated and ADCC is potent. (ii) Potentially, antibodies could bind at non-ideal epitopes, but this may still allow FcγR dimerization, although non-ideally thus preventing full intracellular domain signaling motifs from forming, resulting in partial ADCC. (iii) Some epitopes may present antibody Fc in geometries that physically will not allow FcγR dimerization, thus blocking kinase access and resulting in no ADCC.

Given the above discussions, we are led to the idea that antibody angle-of-approach and Fc presentation may indeed play an important role in how well an antibody performs NK cell ADCC. Many structures of antibodies in complex with viral antigens show that antibody Fab can bind at multiple angles-of-approach (Figure 4) (102). This may suggest that the Fc domains of these antibodies are differentially displayed to the immune system as well. How close antigens are to each other, as well as their individual size and shape, could influence how Fc domains are presented as well as the level of avidity experienced (Figure 11A). There is some evidence to suggest that where an antibody binds on antigen may influence innate effector activity for HIV (261), influenza (262, 263) and Ebola viruses (104, 264), among other examples, but there is not yet convincing molecular data to provide a general model that ties these observations together.

Given the space requirements for receptor signaling to occur between cells, along with the details of structures of both Fab-antigen and Fc-FcγR complexes, there is some suggestion that antibody Fc and upper-hinge must be presented in a specific way in order for FcγRs to recognize them, with the Fc folded over parallel to the effector cell surface (Figures 8B, 9B,C) (2, 12). This provides space for every type of FcγR so far described to dimerize in the extracellular region without clashing with bound Fc (Figure 9B). This type of binding is well-suited to antibody structure, which is a unique ligand because of flexibility between antigen and receptor binding domains provided by hinge-regions. The degree of flexibility can vary depending on subclass and may correlate with FcγR binding and activation activity. In this model, antibodies could be quite effective at assembling the ADCC activation complex over a wide range of antibody binding angles but may be inhibitory in certain situations (Figure 11B). Additionally, this model also provides for the tuning of activation that could result from less than ideal but still permissive geometries of FcγR dimer assembly. However, there are currently no structures of full-length antibody bound to antigen and/or FcγRs that would provide the types of details required to reconcile structures of isolated domains in the context of the IS. This field is ripe for discovery and will benefit from future studies that reflect those already accomplished in the T cell field.

## Next-generation Approaches to Studying NK Cell ADCC

Much of our understanding of NK cell ADCC, as well as antibody effector function in general, is based on individual biochemical studies or fragmented structural biology. Tying these data together is proving to be difficult and is only beginning to paint a picture that seems pervasive throughout immunology, which is that the immune system is dynamic and variable as well as extensively complex. Therefore, many scientists are beginning to rethink altogether how they approach such difficult questions as those regarding antibody effector function. The next wave of impactful research toward defining realistic and meaningful hypotheses about NK cell function and how to design better therapeutics will almost certainly derive from taking a “bigger picture” approach, such as offered from omics-type studies. Below, I will describe what these approaches are and how they are being used to answer questions regarding NK cell activation.

### Transcriptomics, Proteomics, and Metabolomics

The term “omics” refers to the collective, encompassing and complete study of a particular aspect of biology (265). The major contributors to integrative omics techniques, now referred to as “systems biology,” are genomics, transcriptomics, proteomics and metabolomics. These techniques refer to, respectively, the collective and unbiased study of the genes, RNA, proteins and metabolites that make up single cells, tissues or whole organisms (266, 267).

Historically, these fields have been quite niche owing to the immense amount of time, expertise and expense associated with using any one of them for analyzing a biological question. For example, sequencing the first human genome is estimated to have cost $2.7 billion US dollars and took nearly 15 years to complete. However, advances in technology have not only made these techniques much cheaper and faster, but they are now accessible to nearly any scientist in any field. Much of this success has stemmed from advances in computing, automation and bioinformatics that can handle the massive amounts of samples and data needed to be analyzed. Only recently have these techniques found a foothold in the service-based scientific industry as well as core technologies at many academic institutes. We are now beginning to see immunologists exploit omics as a means of hypothesis generation and an exciting new way to tackle the study of disease, donor immune response variability and mining for distinct cellular subsets (268).

### Omics Studies on NK Cell Activation

Historically, NK cells are distinguished by cell surface markers which tend to lump NK cells into two populations as cytotoxic and regulatory, CD56^bright^ and CD56^dim^, respectively (35). However, transcriptomic profiles of NK cells are beginning to demonstrate a wide range of heterogeneity within NK cell populations. For example, one study utilized single cell RNA sequencing (scRNA seq) to determine transitional populations of NK cells within bone marrow and PB that exist between these two examples (269). Another group demonstrated that NK cells exhibit organ-specific transcriptional profiles (270). Overall, these studies indicate an ability of NK cells to adapt to their location and to maintain plasticity during development. Additionally, transcriptomics has been able to distinguish up to 29 different immune cells types based off of their gene expression profiles, offering a useful tool to study NK cell activation in the context of multiple immune cells or within a whole organism (271, 272).

NK cell activation has also been shown to induce unique transcriptional profiles depending on the type of activation stimulus (97). For example, comparison of ADCC, cytokine and direct activation of primary NK cells showed unique gene expression profiles and differential expression of genes commonly associated with NK cell activation (196). Further, HIV infected individuals have NK cells that differ in their activation profiles from healthy donors, indicating that viral infection can alter NK cell activity. This has similarly been demonstrated for CMV infected individuals, where NK cells can adapt over time and act more like an adaptive immune cell (273). These type of RNA seq studies expose the subtleties in NK cell activity that can be distinguished by gene expression data.

Proteomic analysis serves as an important way to understand the relationships to gene expression data. The earliest studies of proteomes in NK cells utilized gel electrophoresis to identify membrane enriched proteins, differences in proteins found in activated vs. resting NK cells as well as the identity of microvesicle enriched proteins (274, 275). As technology has advanced, larger numbers of proteins have been identified in an unbiased manner, demonstrating the growing utility of proteomics (276, 277). A more recent study used proteomic analysis to identify proteins important for NK cell proliferation and pointed a pathway toward increasing the activity of NK cells in a tumor model through therapeutic blockade (278).

Metabolic studies have also revealed a new aspect of NK cell biology and differentiation based on the influence of metabolic factors outside of traditional routes of cellular influence, and these studies have been well-reviewed recently (279–282). Indeed, large differences in metabolic processes have been identified within NK cell subsets. These differences can help distinguish the regulatory, cytotoxic, and memory functions of NK cells. Robust metabolism is essential for efficient cytotoxicity, but metabolic evidence suggests that activated NK cells use alternative routes for oxidative phosphorylation (279, 280). Generally, our knowledge of NK cell metabolism is quite limited and has left many questions unaddressed, such as the role of metabolism in organ specific and tissue resident NK cells or whether metabolism can be used to modulate immunotherapies. Further, this field has not tapped into the robust tools of metabolomics yet, which could provide much broader *in vivo* based knowledge. Although, the field of discovery-based metabolomics is becoming a more accessible technique, it unfortunately still requires large amounts of starting material, which can be inhibitory in certain experimental setups.

## Closing Remarks

So, what does make an effective antibody for recruiting NK cell ADCC? Unsurprisingly, the answer to that question remains incomplete, but what is clear is that fully understanding the underlying mechanisms of antibody effector functions is a complex and difficult task. While this review was certainly not meant to be comprehensive, it was meant to set a stage for understanding the more subtle roles of the molecular underpinnings that seed NK cell activation in the context of ADCC, as well as to provide a perspective from the point-of-view of structural biology. The roles of structural and biophysical constraints entailed in antibody-based cellular activation have historically been overlooked, and subsequently poorly explored and understood. When developing an antibody with therapeutic potential for NK cell recruitment, one must ask many questions beyond simply “what is the target?” For example, where *exactly* does this antibody bind? What does the target *look* like? How will the antibody coordinate activating ligands as a full IgG? How will this interplay with the rest of the IS? How will these factors affect the dynamics and coordination of activation?

These types of considerations are important because we have learned from experience that *in vitro* activity and validation have not always translated well to the clinic. While brute force in evaluating many different potential targets and antibodies is certainly a way to address the gap in treatment for many diseases, a far more cost effective and long-term solution is to rethink how to approach antibody therapeutic research. While comparing autoimmune disease, genetic disorders, cancer and pathogenic infection is difficult to do, the human immune system is designed to be a one stop shop for handling all of these conditions. Therefore, tackling how to harness that power should be, in theory at least, straightforward to do once we gain a better understanding of how it operates.

**Figure 12:**
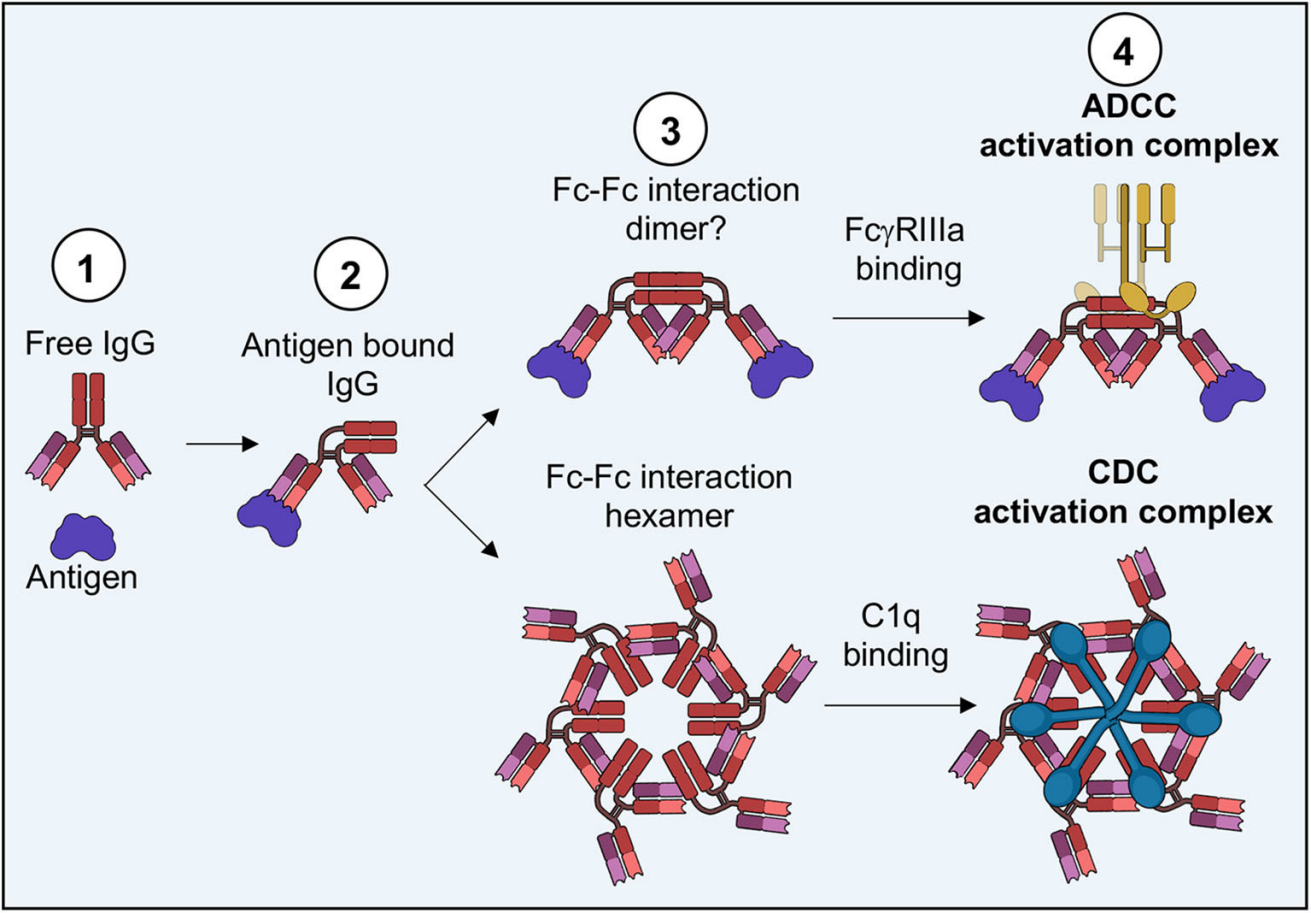
Potential model for IgG effector function fate. (1) Free IgG binds to antigen, (2) causing a change in presentation of the IgG Fc domain. (3) Depending on the arrangement of antigen and/or antibody phenotype, Fc-Fc interactions are facilitated in different oligomeric arrangements. (4) Specific arrangements of Fc-Fc domains influence the binding of FcγRs or C1q, thus resulting in ADCC, CDC or potentially other effector functions.

By returning to the basic biology of NK cell activity, and understanding the molecular nature of activation, we can develop more broadly applicable principles of antibody function and correlates of protection that can be consistently relied upon. Already, the many advances pointed out in this review begin to paint a picture of a more concerted mechanism for how antibodies function post-antigen binding, offering an exciting potential model for antibody effector fate (Figure 12). By combining this simple model with increasing knowledge regarding antibody/receptor glycosylation, as well as the more complex functions of tissue resident immune cells, engineering antibody function may become much more straightforward.

In the future, access to many of the more complex applications discussed here, such as single cell transcriptomics and MINFLUX live cell nanoscopy, may become more commonplace as technology becomes cheaper and computer processing power become more powerful and widely available. With access to such technologies, immunologists can add new layers to their understanding of how molecular perturbations affect higher order cellular functions. This density of information will make understanding antibody function *in vivo* much easier, by building a basis of understanding across biological scales up to the organismal level.

## Author Contributions

The author confirms being the sole contributor of this work and has approved it for publication.

## Conflict of Interest

The author declares that the research was conducted in the absence of any commercial or financial relationships that could be construed as a potential conflict of interest.
